# Defining the Middle Corona

**DOI:** 10.1007/s11207-023-02170-1

**Published:** 2023-06-14

**Authors:** Matthew J. West, Daniel B. Seaton, David B. Wexler, John C. Raymond, Giulio Del Zanna, Yeimy J. Rivera, Adam R. Kobelski, Bin Chen, Craig DeForest, Leon Golub, Amir Caspi, Chris R. Gilly, Jason E. Kooi, Karen A. Meyer, Benjamin L. Alterman, Nathalia Alzate, Vincenzo Andretta, Frédéric Auchère, Dipankar Banerjee, David Berghmans, Phillip Chamberlin, Lakshmi Pradeep Chitta, Cooper Downs, Silvio Giordano, Louise Harra, Aleida Higginson, Russell A. Howard, Pankaj Kumar, Emily Mason, James P. Mason, Richard J. Morton, Katariina Nykyri, Ritesh Patel, Laurel Rachmeler, Kevin P. Reardon, Katharine K. Reeves, Sabrina Savage, Barbara J. Thompson, Samuel J. Van Kooten, Nicholeen M. Viall, Angelos Vourlidas, Andrei N. Zhukov

**Affiliations:** 1 Southwest Research Institute, 1050 Walnut Street, Suite 300, Boulder, CO 80302 USA; 2 Space Science Laboratory, University of Massachusetts Lowell, Lowell, Massachusetts USA; 3 Center for Astrophysics | Harvard & Smithsonian, Cambridge, MA 02138 USA; 4 DAMTP, CMS, University of Cambridge, Wilberforce Road, Cambridge, CB3 0WA UK; 5 NASA Marshall Space Flight Center, Huntsville, AL 35812 USA; 6 New Jersey Institute of Technology, 323 Martin Luther King Jr. Blvd., Newark, NJ 07102 USA; 7 U.S. Naval Research Laboratory, Code 7213, 4555 Overlook Ave. SW, Washington, DC 20375 USA; 8 Mathematics, School of Science & Engineering, University of Dundee, Nethergate Dundee, DD1 4HN UK; 9 Southwest Research Institute, 6220 Culebra Road, San Antonio, TX 78238 USA; 10 NASA Goddard Space Flight Center, Code 670, Greenbelt, MD 20771 USA; 11 ADNET Systems, Inc., Greenbelt, MD 20771 USA; 12 INAF - Osservatorio Astronomico di Capodimonte, Salita Moiariello 16, I-80131 Naples, Italy; 13 Université Paris-Saclay, CNRS, Institut d’Astrophysique Spatiale, 91405 Orsay, France; 14 Indian Institute of Astrophysics, 2nd Block, Koramangala, Bangalore, 560034 India; 15 Solar-Terrestrial Centre of Excellence – SIDC, Royal Observatory of Belgium, Ringlaan - 3 - Avenue Circulaire, 1180 Brussels, Belgium; 16 Laboratory for Atmospheric and Space Physics, Space Science, 3665 Discovery Dr, Boulder, CO 80303 USA; 17 Max-Planck-Institut für Sonnensystemforschung, Justus-von-Liebig-Weg 3, 37077 Göttingen, Germany; 18 Predictive Science Inc., 9990 Mesa Rim Rd, Suite 170, San Diego, CA 92121 USA; 19 INAF-Astrophysical Observatory of Torino, via Osservatorio 20, I-10025 Pino Torinese, Italy; 20 ETH-Zürich, Hönggerberg campus, HIT building, Zürich, Switzerland; 21 Applied Physics Laboratory, Johns Hopkins University, 11100 Johns Hopkins Rd., Laurel, MD 20723 USA; 22 American University, Washington, DC 20016 USA; 23 Department of Maths, Physics and Electrical Engineering, Northumbria University, Newcastle upon Tyne, UK; 24 Embry-Riddle Aeronautical University, 1 Aerospace Blvd., Daytona Beach, FL 32114 USA; 25 NOAA National Centers for Environmental Information, 325 Broadway, Boulder, CO 80305 USA; 26 National Solar Observatory, 3665 Discovery Drive, Boulder, CO 80303 USA; 27 Skobeltsyn Institute of Nuclear Physics, Moscow State University, 119992 Moscow, Russia

**Keywords:** Corona

## Abstract

The middle corona, the region roughly spanning heliocentric distances from 1.5 to 6 solar radii, encompasses almost all of the influential physical transitions and processes that govern the behavior of coronal outflow into the heliosphere. The solar wind, eruptions, and flows pass through the region, and they are shaped by it. Importantly, the region also modulates inflow from above that can drive dynamic changes at lower heights in the inner corona. Consequently, the middle corona is essential for comprehensively connecting the corona to the heliosphere and for developing corresponding global models. Nonetheless, because it is challenging to observe, the region has been poorly studied by both major solar remote-sensing and in-situ missions and instruments, extending back to the *Solar and Heliospheric Observatory* (SOHO) era. Thanks to recent advances in instrumentation, observational processing techniques, and a realization of the importance of the region, interest in the middle corona has increased. Although the region cannot be intrinsically separated from other regions of the solar atmosphere, there has emerged a need to define the region in terms of its location and extension in the solar atmosphere, its composition, the physical transitions that it covers, and the underlying physics believed to shape the region. This article aims to define the middle corona, its physical characteristics, and give an overview of the processes that occur there.

## Introduction

Parker (1958) showed that the hot corona cannot maintain a hydrostatic equilibrium. Instead, the pressure-gradient force exceeds gravity and produces a radial acceleration of the coronal plasma to supersonic velocities: the *solar wind*. Early solar-wind velocity observations by the *Ulysses* spacecraft (see, e.g., Bame et al., 1992) showed that the solar wind was split rather simply between fast and slow components, the fast wind emanating generally from the interiors of (polar) coronal holes and the slow wind originating near the ecliptic plane. Observations frequently deviate from this traditional fast/slow dichotomy, so models of coronal heating and solar-wind acceleration must encompass a much more diverse set of conditions and phenomena to truly achieve a realistic description of the physics of the solar wind (Verscharen, Klein, and Maruca, 2019).

The solar-wind acceleration region was originally thought to originate beyond 10 solar radii [$\mathrm{R}_{\odot }$]; however, new observations suggest that this critical region originates closer to the solar surface (Wexler et al., 2020; Raouafi et al., 2023). This height is dictated by the interplay between the open and closed magnetic fields, their origins and boundaries, as described by open-flux corridors and the S-web (Antiochos et al., 2011; Titov et al., 2011). A new system of source, release, and acceleration mechanisms for solar-wind types characterized beyond the traditional fast–slow wind dichotomy was presented by Viall and Borovsky (2020). Several of those mechanisms (e.g. streamer-blob release) take place at locations within the *middle corona*.

The middle corona is a critical transition region between the highly disparate physical regimes of the inner and outer solar corona. (Throughout this article we will adopt the common nomenclature of *inner* and *outer* corona, as opposed to *lower* and *upper*, or *extended* corona.) Nonetheless, the region remains poorly understood, primarily due to historical difficulties in observing it. The boundaries of the region have been debated for many years. Nevertheless, through a series of open community meetings and extensive discussions, we have arrived at a common set of boundaries to define the middle corona. Our consensus considers both the variation in roles that different physical mechanisms play throughout the corona and the historical observational context of coronal observations. We define the middle corona as ≈ 1.5 – 6 $\mathrm{R}_{\odot }$ (measured from disk center).

The inner boundary roughly traces the tops of the closed magnetic-field structures that dominate the inner corona, below which loops appear and hydrostatic scale heights are often applicable (e.g. Koutchmy and Livshits, 1992; Koutchmy and Molodensky, 1994; Winebarger et al., 2002; Koutchmy, 2004). The outer boundary is roughly pinned to where the solar atmosphere is believed to have *fully* transitioned to an outflow regime, and it is observed to be fully radial in structure. This is evident in coronal-hole structures, which appear to be purely radial beyond 3 – 4 $\mathrm{R}_{\odot }$ and no longer exhibit super-radial expansion (DeForest et al., 1997; DeForest, Lamy, and Llebaria, 2001). Schatten, Wilcox, and Ness (1969) chose the source-surface height for potential-field source-surface (PFSS: see also Wang and Sheeley, 1992) extrapolations based on matching the interplanetary magnetic field to the number of surviving field lines; a value that has typically been located between 3 and 6 $\mathrm{R}_{\odot }$ (e.g. McGregor et al., 2008). It is also around this height that “Sheeley Blobs”, small-scale density inhomogeneities frequently observed flowing both inwards and outwards in streamers, are believed to be pinched-off through magnetic reconnection (e.g. Sanchez-Diaz et al., 2017).

The region thus encapsulates several important physical transitions, including the change from predominantly *closed* to *open* magnetic-field structures, and the change from low to high plasma-$\beta $ in *quiet-Sun* regions (Vourlidas et al., 2020). A list of transitions occurring in this region can be found in Table 1; several inner coronal transitions are also included for comparison.

**Table 1:** Transitions in the inner and middle corona, where: FSW = Fast Solar Wind, SSW = Slow Solar Wind.

Type of transition	Inner corona	Middle corona	Context
Structure		Closed-to-open magnetic-field configurations	SSW, streamer regions
		Density structures/“blobs” released into outflow	SSW, streamer cores
	Confinement regime with elevated density power law radial dependence	Density radial dependence drops to near inverse-square scaling	SSW, streamer regions
Dynamics		Subsonic-to-supersonic solar-wind outflow	SSW
		CME main acceleration and initial shock formation	CME
Plasma physics	plasma-*β* ≪ 1		Coronal Holes & FSW
	plasma-*β* < 1 in innermost corona	Broad range of *β* spanning < 1 to > 1	SSW, streamer regions
	Charge state freeze-in		FSW
		Stabilization/freeze-in of ionization charge states	SSW
	Gravitational settling affecting FIP abundances		Streamer bases
		Gravitational settling affecting FIP abundances	Streamer cores
	Coulomb collisions to kinetic plasma processes		FSW
		Coulomb collisions to kinetic plasma processes	SSW

New observations reported by Seaton et al. (2021) suggest heliospheric solar-wind structures not only originate in the inner corona (e.g. DeForest et al., 2018), but can originate from complex dynamics in the middle corona (Chitta et al., 2023). The region is also believed to influence the inner corona, where downflows have been shown to interact with structures below. For example, supra-arcade downflows (SADs: e.g. Savage, McKenzie, and Reeves, 2012; Shen et al., 2022) observed in the wake of eruptions correspond to plasma pile up in the inner corona, and smaller or fainter downflows may also be ubiquitous in the less dynamic atmosphere (Sheeley and Wang, 2002). Such downflows may trigger larger scale eruptive phenomena, or erode magnetic fields that could trigger eruptions through mechanisms such as magnetic breakout (e.g. Antiochos, DeVore, and Klimchuk, 1999). Thus, the middle corona not only plays an important role in shaping outflow, as the region through which all outflows and eruptions must pass and be modulated, but the middle-corona’s physics also has important implications for unified coronal-heliospheric models.

**Figure 1:**
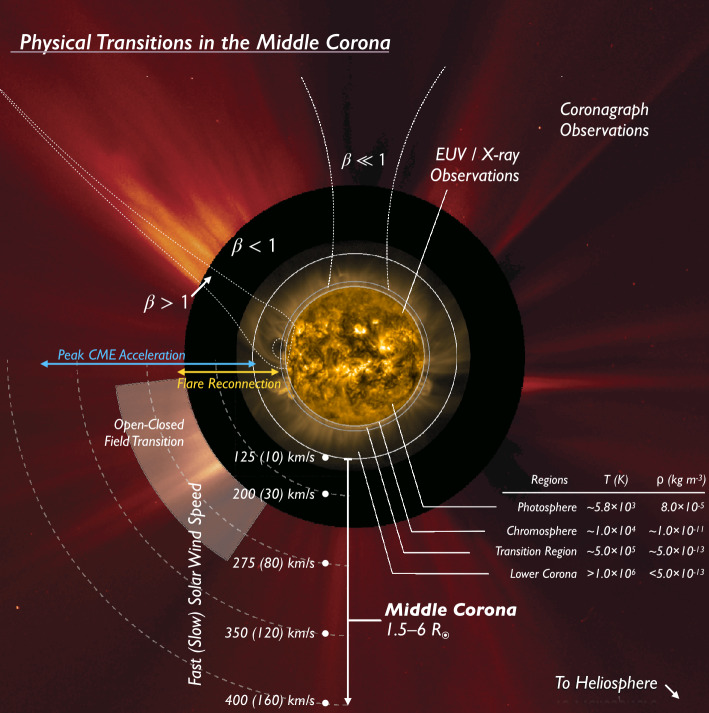
A SWAP and LASCO composite image highlighting the middle corona, and the physical transitions that extend through the region. The image also highlights the observational gap between EUV observations of the inner corona and visible-light observations of the outer corona, currently experienced from the Earth perspective (e.g. Byrne et al., 2014). The image is annotated to highlight key heights, coronal characteristics, and physical transitions.

Historically, the solar corona and its continuous evolution have most commonly been studied using a combination of extreme ultra-violet (EUV) and X-ray observations of the inner corona with visible-light coronagraph observations of the outer corona, as shown in Figure 1. The observations in the figure are from 2014, when the Sun was near the peak of its activity cycle, and they include an EUV image in the center (gold false color), from the large field-of-view (FOV) PROBA2/*Sun Watcher with Active Pixels and Image Processing* (SWAP: Seaton et al., 2013b; Halain et al., 2013) imager, whose passband is centered on 17.4 nm, and a visible-light image (red false color) from the *SOlar and Heliospheric Observatory* (SOHO: Domingo, Fleck, and Poland, 1995)/*Large Angle and Spectrometric Coronagraph* (LASCO: Brueckner et al., 1995) C2 coronagraph, around the edge.

Figure 1 is annotated to highlight atmospheric regions, phenomena, characteristic solar-wind speeds, and various coronal transitions. The EUV observations of the inner corona reveal the shape of structures permeating the region – highlighted by the emitting plasma – that are constrained by the corona’s magnetic field. EUV observations of this region reveal it to be largely dominated by closed magnetic structures. In contrast, the visible-light observations reveal more striated structures, indicative of open magnetic structures, extending into the heliosphere.

Although EUV and visible-light observations have both served as synoptic probes of the corona, these two observational regimes have generally been focused on different regions of the middle corona, and through disparate passbands. Thus, they capture different physical characteristics of the underlying plasma: emission measure within a specific temperature range in the case of EUV and temperature-independent electron density in the case of visible light.

The general lack of continuously available overlap between the different methods of observation, especially from the Earth’s perspective (highlighted by the observational gap in Figure 1) can lead to ambiguity, both when tracking structures and inferring plasma properties such as temperatures and densities. Methods to continuously infer plasma properties include extrapolation and modeling (e.g. Lynch, 2020; Schlenker et al., 2021); however, even for the relatively simple case of a quiet-Sun-streamer structure, various different estimates of the densities and temperatures have been published (Del Zanna et al., 2018). To fully elucidate the mechanisms affecting the large-scale structural and dynamic changes occurring over the middle corona, complementary observations that overlap adjacent zones are essential.

In this article, we propose a definition for the region called the middle corona, we review how we observe it and what we know about it, and we present both the open questions concerning the region and a strategy to explore it. In Section 3 we describe how we currently – and historically – observe the middle corona; in Section 4 we describe the properties and topology of the middle corona; in Section 5 we describe some of the efforts to model and extrapolate properties of the region. Finally, in Section 6 we present a discussion of the region in the form of open questions pertaining to the region and ways of answering them.

## Partitioning the Solar Atmosphere

Although the Sun is effectively a continuous ball of plasma with no physical boundaries, the solar interior is typically demarcated into layers based on the dominant physical processes that govern the energy transport in the respective regions. A similar logic is applied to the solar atmosphere, where the partitions are based on thermal and magnetic properties. These properties not only dictate the emission mechanisms and physical length-scales at play, but ultimately how we observe and model the different regions.

In general, the high magnetic-field strengths, plasma conductivity, temperatures, and densities, and the inhomogeneity of these properties within the inner corona, make formal calculations of its properties inherently complex, so the average properties of particles are often adopted. This introduces the magnetohydrodynamic (MHD) approach to modeling the region, which treats the plasma as a bulk magnetized fluid (Gombosi et al., 2018, and references therein). In the outer corona, where length-scales have increased, kinetic models are both more practical and more commonly used, and the equations of motion for each particle, subjected to various forces, are calculated (Marsch, 2006, and references therein). The middle corona acts as the interface between these two regions and therefore requires a combination of approaches.

The transitions between the three very distinct physical regimes of the inner, middle, and outer corona are not themselves distinct, largely due to the range in length-scales and scale heights experienced among different coronal regions (Chhiber et al., 2022; Malanushenko et al., 2022), and their variation throughout the solar cycle (Badalyan, Livshits, and Sykora, 1993; Edwards et al., 2022). However, rapidly advancing observational and data-processing techniques have provided new insights into the region, and new proposed missions to explore the region have led to the term “middle corona” entering the solar and heliospheric physicists’ lexicon in recent years (e.g. Koutchmy, 2004). There is a clear need to define both the terminology describing this region as well as its properties, which is the goal of this article.

## How We Observe the Middle Corona

**Figure 2:**
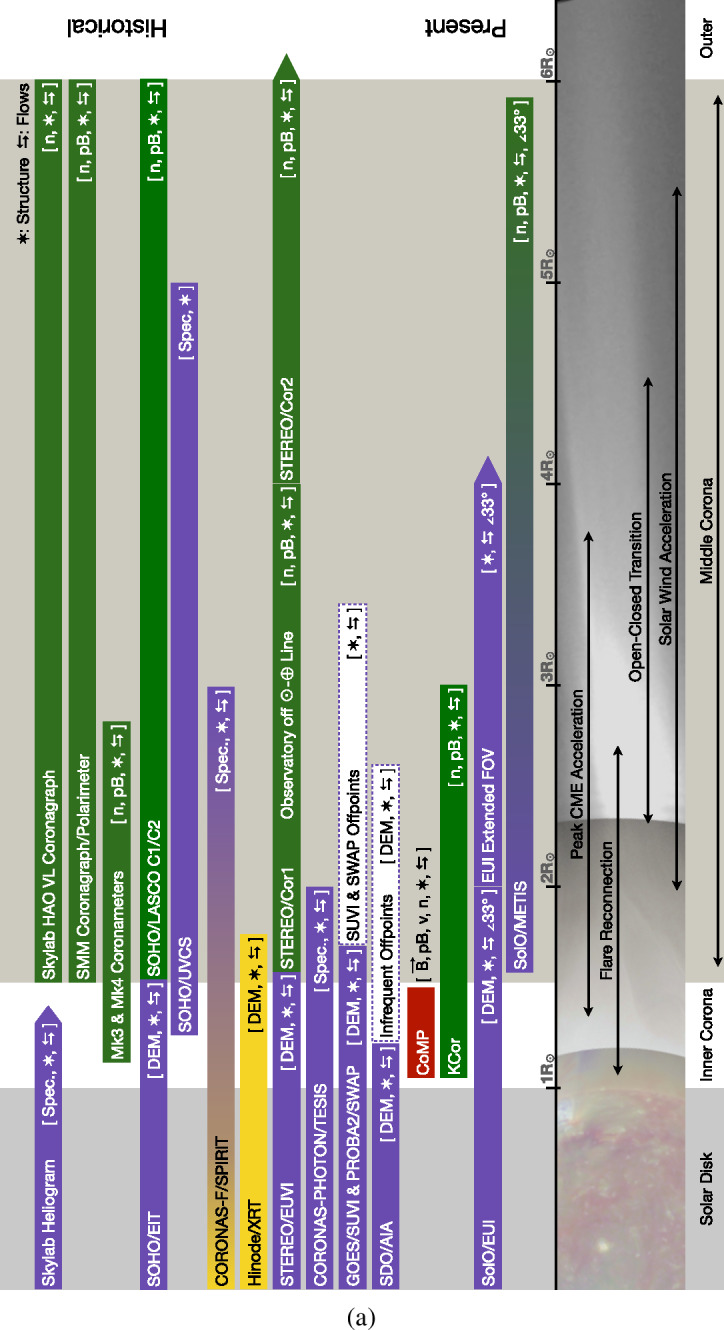

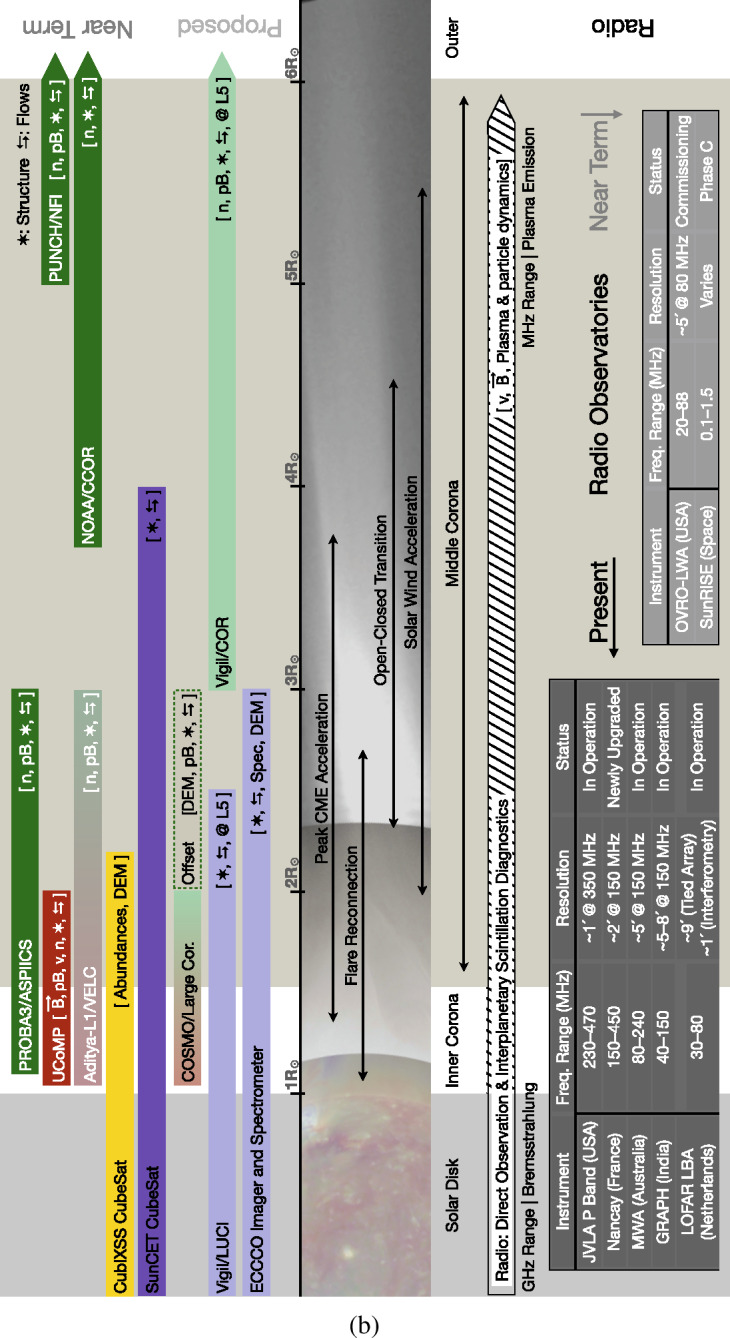

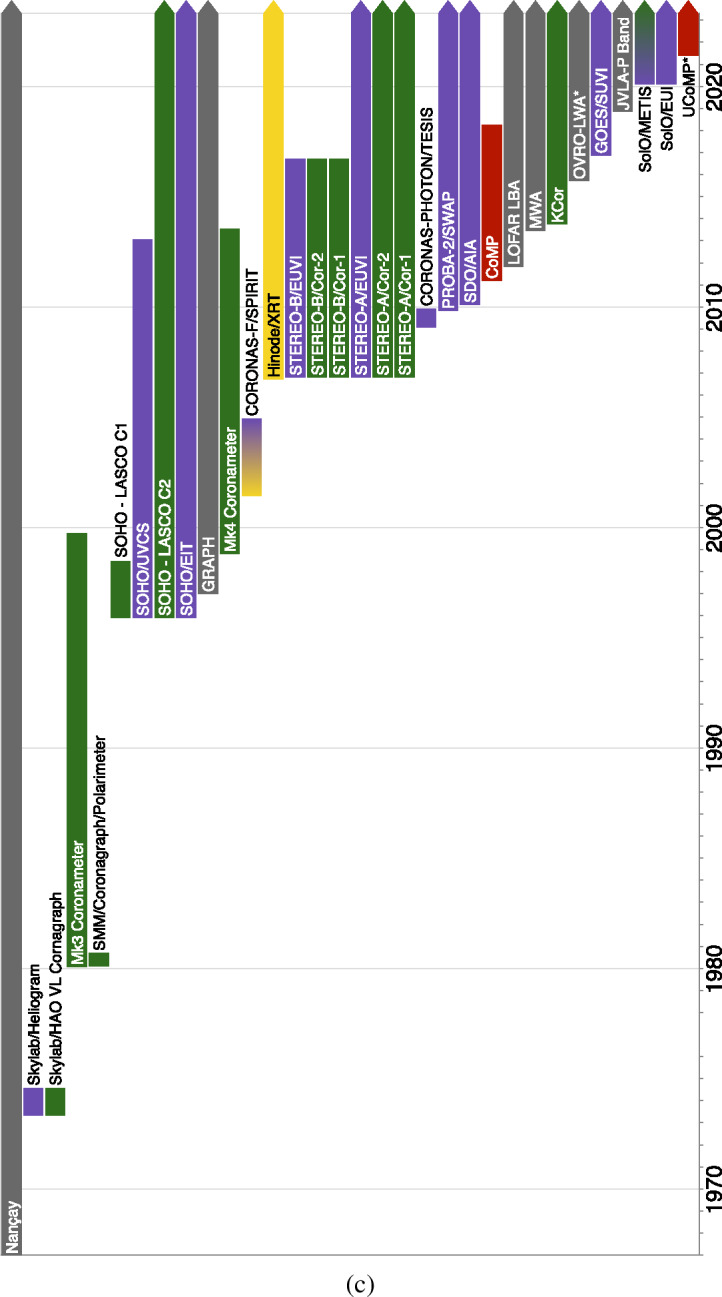
(**a**) Summary of past, present, planned, and proposed middle-corona observatories. The type of observation is indicated in parentheses, with key to symbolic abbreviations in *upper right* of the figure. Color corresponds to the wavelength regime of the observation, X-ray (*Gold*), EUV/UV (*Violet*), Visible (*Green*), Infrared (*Red*), and Radio (*Gray*). (**b**) Continuation of middle-corona observatories. The type of observation is indicated in parentheses, with key to symbolic abbreviations in *upper right* of the figure. Color corresponds to the wavelength regime of the observation, X-ray (*Gold*), EUV/UV (*Violet*), Visible (*Green*), Infrared (*Red*), and Radio (*Gray*). (**c**) Continuation of middle-corona observatories. Past and present instrumentation in order of first light. *Color corresponds to the wavelength regime of the observation, X-ray (Gold), EUV/UV (Violet), Visible (Green), Infrared (Red), and Radio (Gray)*.

There are a variety of reasons that the middle corona has not been as well characterized as other regions of the solar atmosphere. These include limitations on instrumentation and instrumentation capabilities, prioritization of other investigations, and the observations of other regions. Nonetheless, through dedicated observation campaigns and increasingly sophisticated spectroscopic, imaging, and data-processing techniques, large portions of the middle corona have been intermittently probed. Figure 2 presents a rough overview of many past, present, planned, and proposed observatories that contribute to our knowledge of the region.

Many of the most prominent observations of the middle corona have been made in wavelengths ranging from X-rays to infrared, but radio imaging and radio measurements of the middle corona have provided important insights into the underlying plasma characteristics. Spectroscopic instruments, particularly in the ultraviolet, have also made important contributions to our understanding of the properties and dynamics of middle-corona plasma.

In general, instruments that make continuous observations in visible-light, EUV, and X-ray passbands are located on space-based platforms, where they can observe the corona unencumbered by the Earth’s atmosphere and day–night cycles, whereas observations at radio wavelengths are made from ground-based sites due to the size of instruments. Both sets of observation utilize different observing techniques, and rely on different emission mechanisms, which we review below. The following section is divided in to two main subsections: The first examines observations made through IR, visible-light, EUV, and X-ray wavelengths (Section 3.1), and the second covers observations made through radio imaging and measurements (Section 3.2).

### Short Wavelengths: Infrared, Visible, UV, and X-Rays

**Figure 3:**
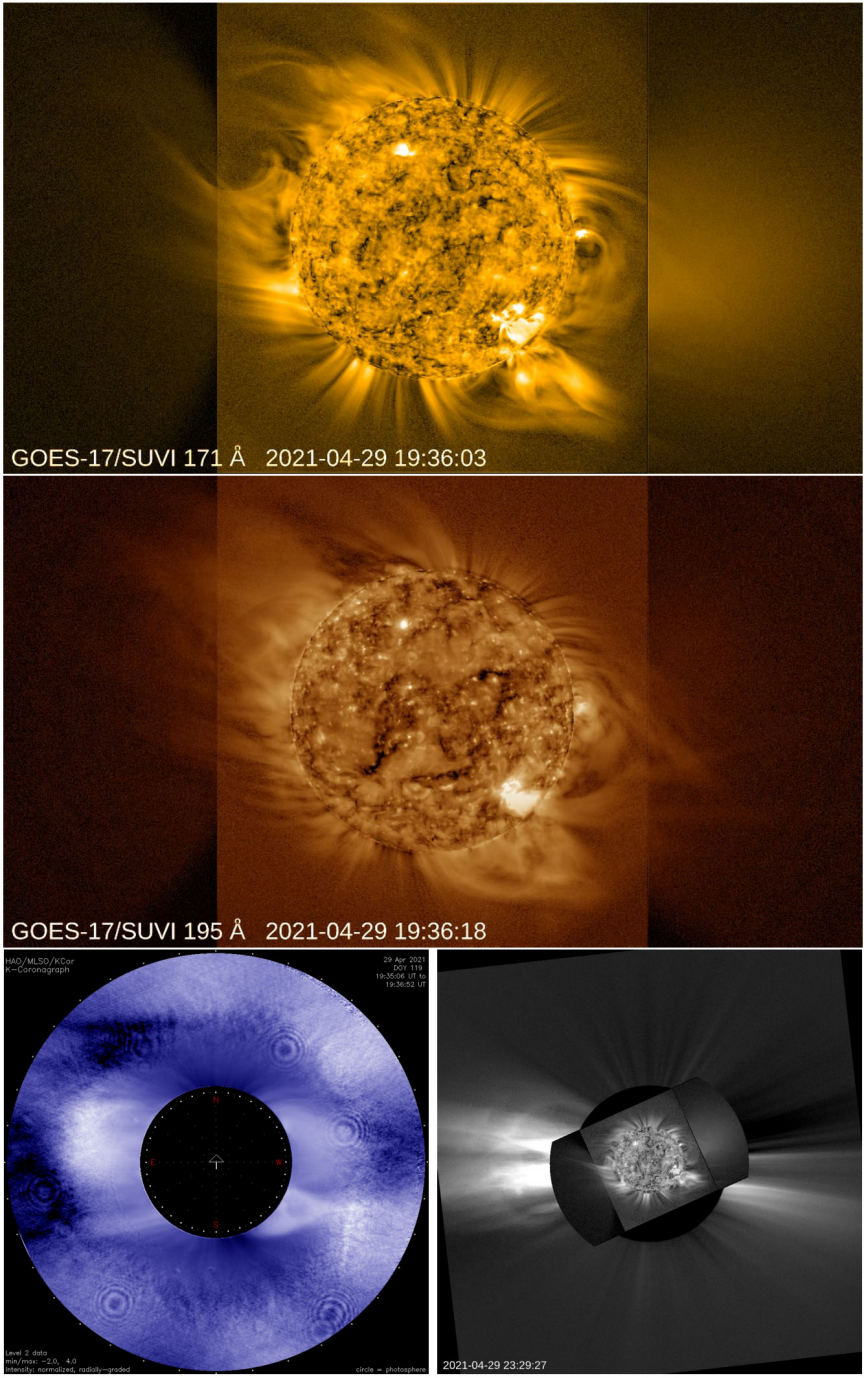
Different views of the middle corona, observed on 29 April 2021, in EUV from SUVI (*top* in 17.1 nm, and *middle panel* in 19.5 nm) and visible light from K-Cor (*bottom left*) and LASCO (*bottom right*; with SUVI superimposed). Images are in camera coordinates and not necessarily co-aligned, although solar North is generally upwards in each frame.

Although there remain persistent observational gaps (Byrne et al., 2014), the middle corona has occasionally been observed by a disparate set of instruments in passbands that range from the infrared to X-ray. The most extensive observations have been made with visible-light images, both from coronagraphs and eclipses, as well as direct EUV imaging, primarily through dedicated off-point campaigns by imagers designed to observe the inner corona. Figure 3 shows examples of several such observations, from a coordinated campaign during April 2021, that included offpoints by the GOES *Solar Ultraviolet Imager* (SUVI: Darnel et al., 2022), the Mauna Loa Solar Observatory’s *K-coronameter* (K-Cor: Elmore et al., 2003), and LASCO on SOHO. These coordinated observations allow us to characterize different aspects of the middle corona, leveraging several different mechanisms through which plasma in the region manifests itself. Here, we provide a brief overview of the history of these observations and the variety of phenomena observed here using these approaches.

#### Observed Emission Mechanisms

The inner corona exhibits temperatures ranging between $T\approx 5\times 10^{5}$ and ${>}\,2 \times 10^{7}\text{ K}$, and consequently highly ionized atoms, emitting at UV, EUV, and X-ray wavelengths, provide a key diagnostic of temperature and are very commonly used to observe this region. This highly ionized emission is strongly dependent on electron density [$n_{ \mathrm{e} }$]. The dominant emission mechanisms are spontaneous emission following *collisional excitation* and *resonant scattering* of incident light by ions. The intensity of emission resulting from scattering mechanisms is proportional to number density [$\propto n_{ \mathrm{e} }$], while emission from collisional excitation is proportional to density squared [$\propto n_{ \mathrm{e} }^{2}$]. In the innermost corona, collisional excitation dominates all emission mechanisms other than broadband Thomson scattering, and in the absence of large-scale structures, its ${\propto}\,n_{ \mathrm{e} }^{2}$ relationship gives rise to a rapid drop-off in brightness as density decreases with height. The belief that this drop-off would limit the viability of EUV observations above 1.5 $\mathrm{R}_{\odot }$ led most past observational efforts at these wavelengths to focus only on the inner corona.

**Figure 4:**
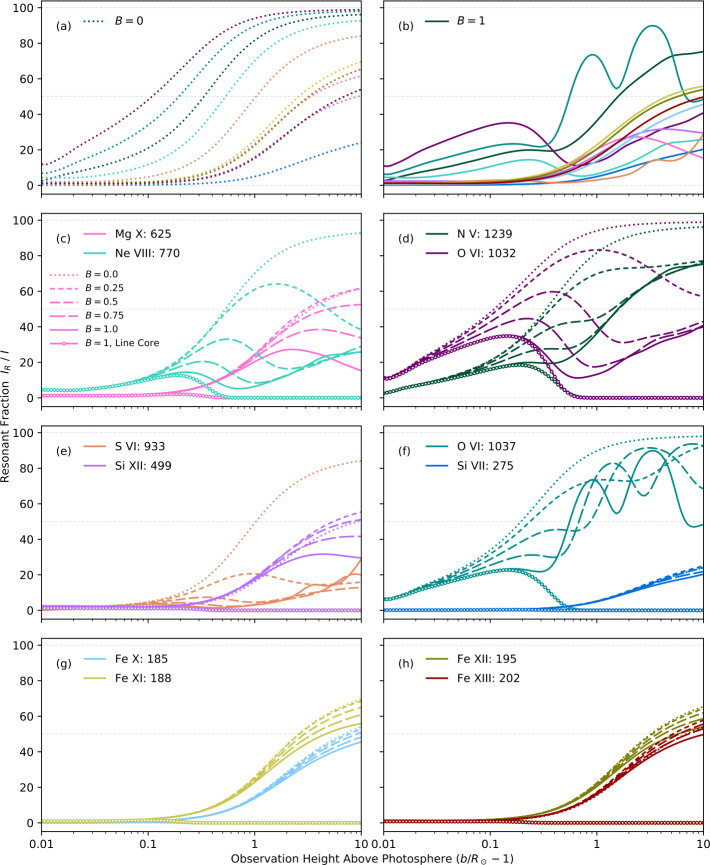
The proportion of the total emissivity contributed by resonant scattering as a function of height, for various fractions [$B$] of the model value of the solar-wind speed, as reported in Gilly and Cranmer (2020). $B$ is a scalar factor applied to the radial-wind-speed profile, with $B=0$ indicating no wind and $B=1$ indicating wind at nominal modeled values. Ion-line wavelengths are given in units of angstroms. (Figure 17 of Gilly and Cranmer, 2020, used with permission).

At larger heights, resonant scattering can begin to dominate the ion and neutral emission. The relative contribution of resonant scattering and collisional excitation to the total emissivity of the plasma depends on the local density (of both ions and electrons), temperature, the collisional-excitation rate, and the incident radiation at a given wavelength. The resonant scattering generally increases the emission, but for some lines, those excited by radiation from particularly strong chromospheric emission lines, Doppler-dimming can lead to a strong decrease of the amplitude of the scattered radiation. As the solar wind leaves the inner corona, it is accelerated until it reaches such a velocity that the incident light is no longer at the same wavelength as the spectral line at rest, thereby reducing the total amount of photon scattering. Note that in addition to the bulk-outflow velocities, there are large thermal motions of the ions (with a dependence on atomic mass) that effectively smear out the relative velocities with respect to the solar surface and reduces the amplitude of the dimming. Occasionally, the scattering can lead to Doppler-pumping, where a Doppler shift causes the resonant wavelength of the coronal ions to match the wavelength of a spectrally adjacent line, such as is the case with O vi 103.8 nm. Figure 4, from Gilly and Cranmer (2020), highlights the relative proportion of resonant scattering to the total emissivity as a function of wind speed, pointing to a potential diagnostic for solar-wind acceleration in the middle corona.

New EUV observations (e.g. Goryaev et al., 2014; Seaton et al., 2021), have shown that resonant scattering of emission from EUV-bright inner-corona features ($\propto n_{ \mathrm{e} }$) occurs in many structures. Thus the brightness of the EUV corona declines less precipitously than anticipated for purely collisionally excited emission. This resonantly scattered emission can enhance the visibility of large-scale features in the middle corona for a new class of coronal observatories (see Section 3.1.2).

In contrast to emission-line diagnostics, broadband visible-light observations from coronagraphs and eclipses reveal Thomson-scattered emission (Inhester, 2015), which is only sensitive to electron density. Differences in the nature of complex 3D structures that are manifested in these different emission mechanisms cannot always be reconciled in multi-wavelength studies. Thus, visual confusion in the fine structure of this region has led to additional barriers to resolving key questions about the middle corona.

In narrow-band visible and near-infrared, because of the much greater flux from the photospheric radiation, resonant scattering begins to dominate the emission processes for lines in this spectral interval already at relatively low heights above the surface. Because of the scattering of that same photospheric emission in the Earth’s atmosphere, and the near lack of space-borne coronagraphs capable of observing coronal emission lines, it is more typical to study the visible-light corona at these wavelengths, both at eclipses and using a polarization-discriminating coronagraph (but see Ding and Habbal, 2017, for emission-line measurements at 2 $\mathrm {R}_{\odot }$).

#### Optical Observations

Images of the middle corona are primarily produced in visible and infrared light using coronagraphic instruments or during eclipses, or in UV, EUV, and X-ray passbands using telescopes that can directly image the solar disk. High-quality eclipse observations that include the middle corona date to the nineteenth century (Holden, 1894), while coronagraphic observations extend the pioneering work of Lyot (1939).

The space age opened the door to both higher quality coronagraphic observations and exploration in EUV and X-rays. An important milestone was the *Skylab* mission, which carried both the *High Altitude Observatory White Light Coronagraph* (MacQueen et al., 1974), whose FOV covered the middle corona more or less exactly – 1.5 to 6.0 $\mathrm{R}_{\odot }$ – and the Naval Research Lab’s *Extreme Ultraviolet Spectroheliograph*, which made spectrally dispersed images of the inner and occasionally middle corona over a wide range of EUV wavelengths (Tousey et al., 1973, 1977). An even more significant breakthrough in middle-corona studies came with the *Solar Maximum Mission* (SMM) *Coronagraph/Polarimeter* instrument (MacQueen et al., 1980), which shared significant heritage and its FOV with the *Skylab* coronagraph, but made many more systematic observations.

These space-based, visible-light observations were augmented by a more sporadic set of ground-based observations: numerous eclipses, coronagraphs such as HAO’s at Climax, Colorado (Wlérick and Axtell, 1957) and on Mauna Loa (*Mark III K-coronameter*; Mk3: Fisher et al., 1981) and several subsequent improved designs. These visible-light instruments and their space-based counterparts exploit the scattering of photospheric light by electrons in the corona (Thomson scattering) to image the corona, and must contend with the challenge of eliminating light from the photosphere, which is nearly $10^{7}$ times brighter than the corona at 1.5 $\mathrm{R}_{ \odot }$. Coronagraphic imaging therefore requires very efficient stray-light suppression, which must overcome both scattering of light and diffraction at the edges of optical components. This is generally easier to achieve with instruments having large separations between occulter and primary objective, in the case of externally occulted instruments, although the specifics of the design of these instruments differ considerably. The challenge of observing close to the solar limb is particularly acute, and, as a result, coronagraphic observations of the innermost middle corona are generally affected by stray light.

Nonetheless, in the more than 25 years since the beginning of the SOHO mission, most of the middle corona has been observed in visible light by the LASCO suite (Brueckner et al., 1995) and in the UV/EUV by the *Ultraviolet Coronagraph Spectrometer* (UVCS: Kohl et al., 1995), in EUV in the far edges and corners of images from the *Extreme-ultraviolet Imaging Telescope* (EIT: Delaboudinière et al., 1995), and subsequently by a fleet of instruments that observed in visible light, EUV, and X-rays, including from multiple perspectives. These include the EUV/Visible *Sun-Earth Connection Coronal and Heliospheric Investigation* (SECCHI) on the twin STEREO spacecraft (Howard et al., 2008), the *SPectrographIc X-ray Imaging Telescope* spectroheliograph (SPIRIT: Zhitnik et al., 2002), the *TElescopic Spectroheligraphic Imaging System* telescope (TESIS: Kuzin et al., 2009), the SWAP *EUV Imager* on PROBA2 (Seaton et al., 2013b; Halain et al., 2013), the GOES *Solar Ultraviolet Imager* (SUVI: Darnel et al., 2022), the GOES *Soft X-ray Imager* (SXI: Hill et al., 2005; Pizzo et al., 2005), the *Hinode/X-Ray Telescope* (XRT: Golub et al., 2007), the *Solar Orbiter/Extreme Ultraviolet Imager* (EUI: Rochus et al., 2020) and the *Metis* coronagraph (Antonucci et al., 2020). Additional planned missions will soon push the boundaries of observations of the middle corona both farther outwards (in EUV) and inwards (for coronagraphs).

**Figure 5:**
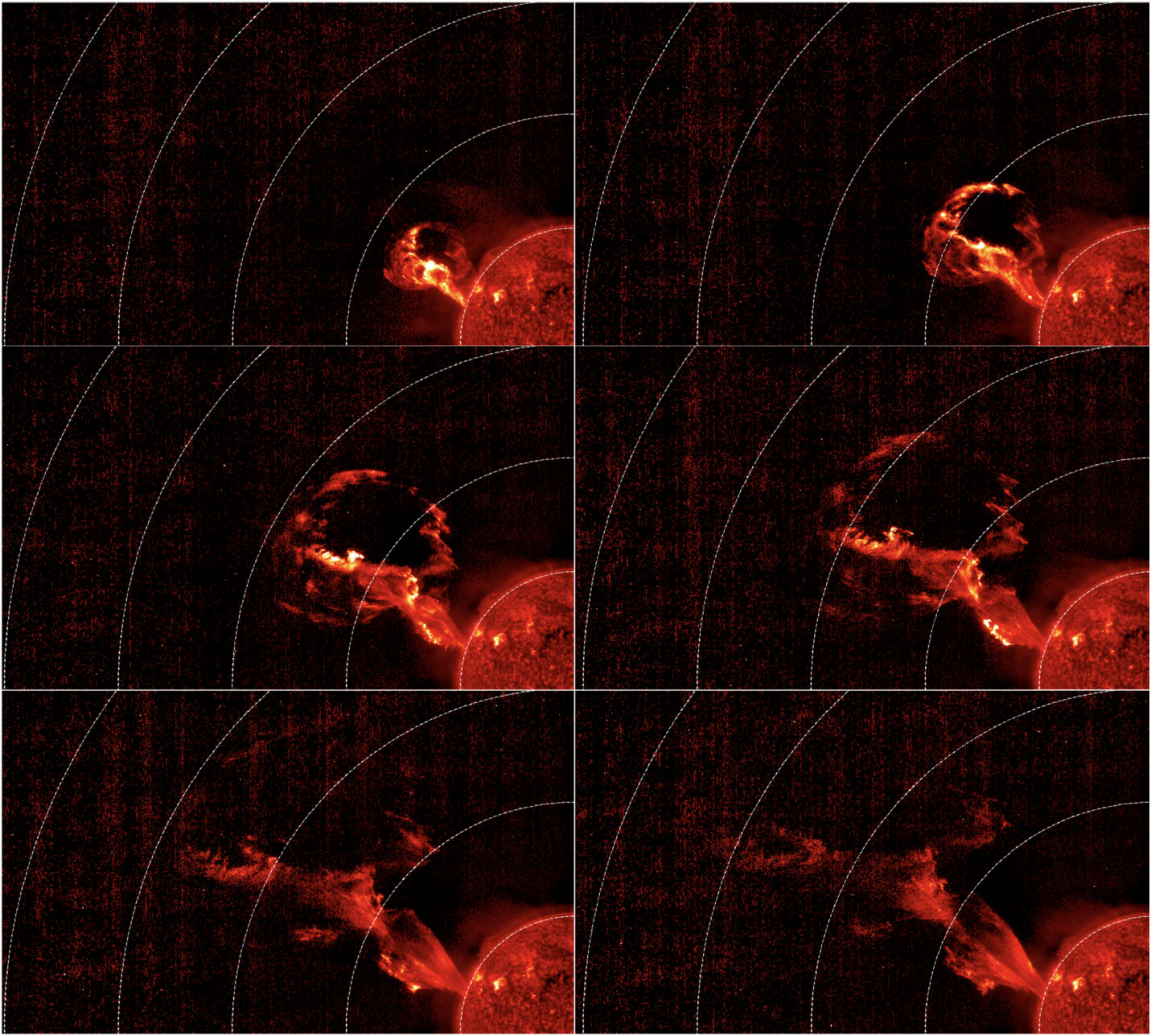
A prominence eruption observed through the 30.4 nm passband of the EUI *Full Sun Imager* on 15 February 2022, when the *Solar Orbiter* spacecraft was located at 0.73 AU from the Sun, at 22:00 UT (*top left*), 22:04 UT (*top right*), 22:10 UT *(middle left*), 22:14 UT (*middle right*), 22:20 UT (*bottom left*), and 22:24 UT (*bottom right*). The observations have been processed using the radial-filtering technique described by Seaton et al. (2023) to enhance the off-limb signal, allowing the eruption to be tracked out to 5 $\mathrm{R}_{\odot }$. See Mierla et al. (2022) for further details about this event.

Arguably the most important innovation in middle-corona studies of the last decade has been a series of exploratory campaigns using off-pointed EUV images. These include both short-term campaigns with the SWAP imager (O’Hara et al., 2019; Goryaev et al., 2014) and long-term campaigns using SUVI (Seaton et al., 2021; Chitta et al., 2023). Such observations, using instruments with medium fields of view in novel ways to extend their observational range – along with a handful of reports from lesser-known instruments with dedicated larger fields of view (e.g. Reva et al., 2017) – definitively proved the feasibility of middle-corona observations with dedicated EUV instruments. The most recent and prominent of these instruments is the *Full-Sun Imager* (FSI) in *Solar Orbiter*’s EUI suite, with a varying instantaneous FOV due to its highly variable distance from the Sun. Early FSI observations have already demonstrated its ability to track erupting prominences from their genesis to the outer edge of the middle corona (Mierla et al., 2022). Figure 5 shows the propagation of such a prominence observed by the FSI on 15 February 2022; the observations have been processed using the radial-filtering technique described by Seaton et al. (2023) to enhance the off-limb signal, allowing the eruption to be tracked out to 5 $\mathrm{R}_{\odot }$.

These pioneering EUV instruments have paved the way for a new generation of EUV instruments and techniques that focus specifically on the middle corona, including the *Sun Coronal Ejection Tracker* (SunCET) CubeSat (Mason et al., 2021, 2022), in development now, and the proposed *EUV CME and* *Coronal Connectivity Observatory* (ECCCO; previously referred to as the *COronal Spectrographic Imager in the EUV* or COSIE: Golub et al., 2020) and a potential successor to the *Lagrange eUv Coronal Imager* (LUCI: West et al., 2020) on the *Vigil* mission.

Likewise, pioneering visible and near-IR observations both from coronagraphs and eclipses have paved the way for a new generation of coronagraph instruments with improved imaging capabilities in the inner and middle corona. These include the *Coronal Solar Magnetism Observatory* (COSMO: Tomczyk et al., 2016), a suite of ground-based coronagraphic instruments, and the *Association of Spacecraft for Polarimetric and Imaging Investigation of the Corona of the Sun* (ASPIICS: Lamy et al., 2010; Galano et al., 2018; Shestov et al., 2021), the visible coronagraph on the PROBA-3 formation-flying space mission. See Figure 2 for a summary of notable historical, active, planned, and proposed middle-corona observations.

Middle-corona studies have also benefited from the development of advanced image-processing techniques during the past two decades. The steep gradient in intensity as a function of height in the corona, both in visible and shorter wavelength observations, means that the dynamic range of solar images is far greater than can be captured in a single exposure by typical scientific cameras or displayed on a computer screen. Therefore, techniques that can overcome this to generate high-quality, large-FOV images, which still preserve fine details on many scales have been developed. There are over 20 separate methods in the literature that process solar imagery to draw out hidden detail (e.g. Druckmüllerová, Morgan, and Habbal, 2011; Seaton et al., 2023; Auchère et al., 2023).

**Figure 6:**
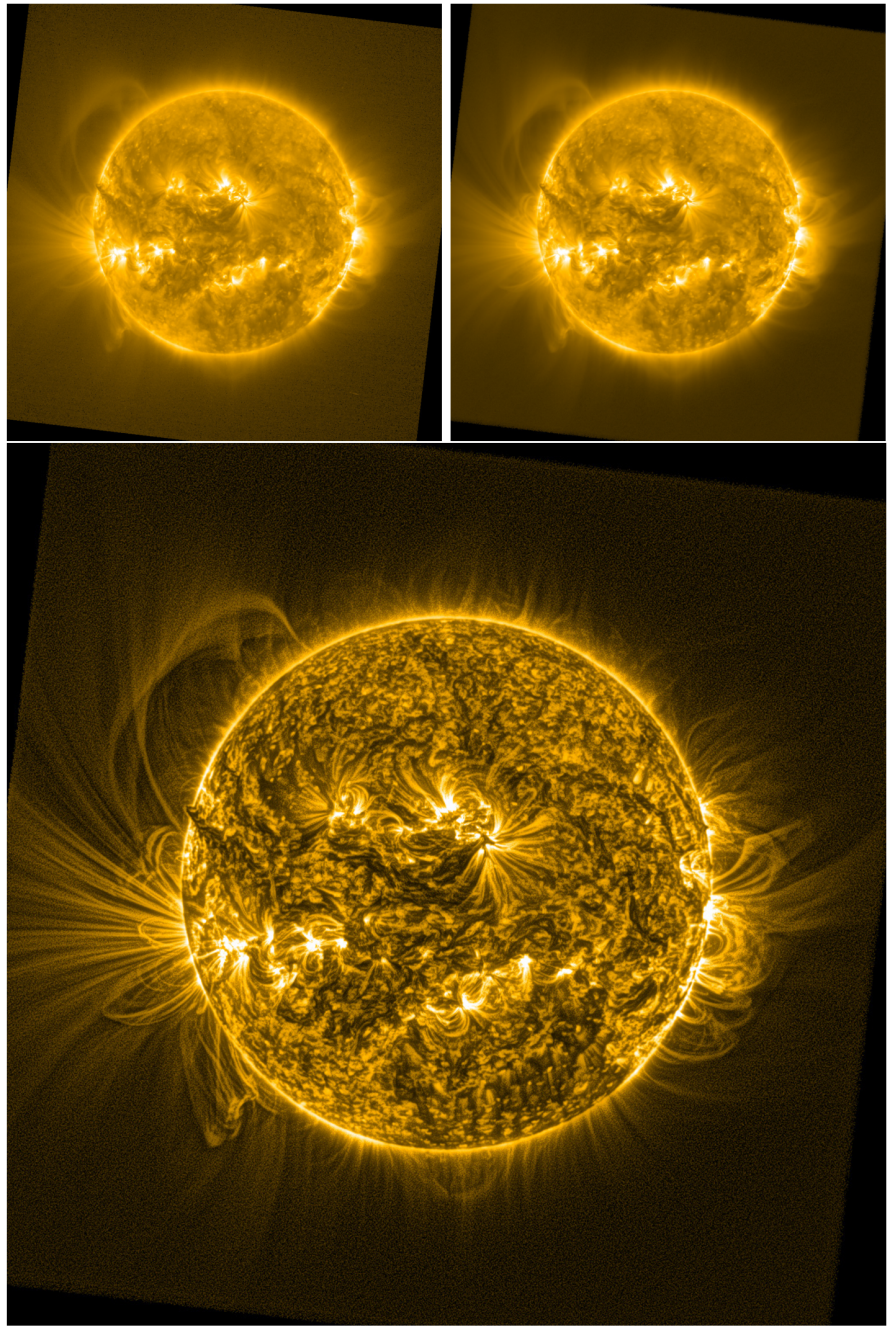
An example of how large FOV images can be processed to reveal structures extending into the middle corona. The three images show the same SWAP (17.4 nm) observation from 10 November 2014, processed nominally (*top left*), using a stacking technique (*top right*, see West et al. (2022) for further details), and using the MGN technique (*bottom*: Morgan and Druckmüller, 2014).

Historically, such dynamic-range challenges were addressed with radially varying optical filters (Newkirk and Lacey, 1970; Eddy, 1989). Contemporary imaging techniques include the stacking of multiple short-exposure observations to approximate a long exposure (e.g. West et al., 2022), and the use of detectors with locally variable exposure times (Mason et al., 2022). Post-processing techniques, which improve the display of these high-dynamic-range images, include computational radial-graded filters (e.g. Martinez, 1978; Seaton et al., 2023), wavelet-based techniques (Stenborg, Vourlidas, and Howard, 2008) and Multiscale Gaussian Normalization (MGN) (e.g. Morgan and Druckmüller, 2014). Figure 6 shows a SWAP EUV 17.4 nm image from 10 November 2014 (top left) and a high-dynamic-range stacked image from the same time (top right) with improved noise characteristics in the outer FOV. The bottom image shows how image processing with the MGN technique can improve the visibility of finer structures.

#### Spectroscopy

**Figure 7:**
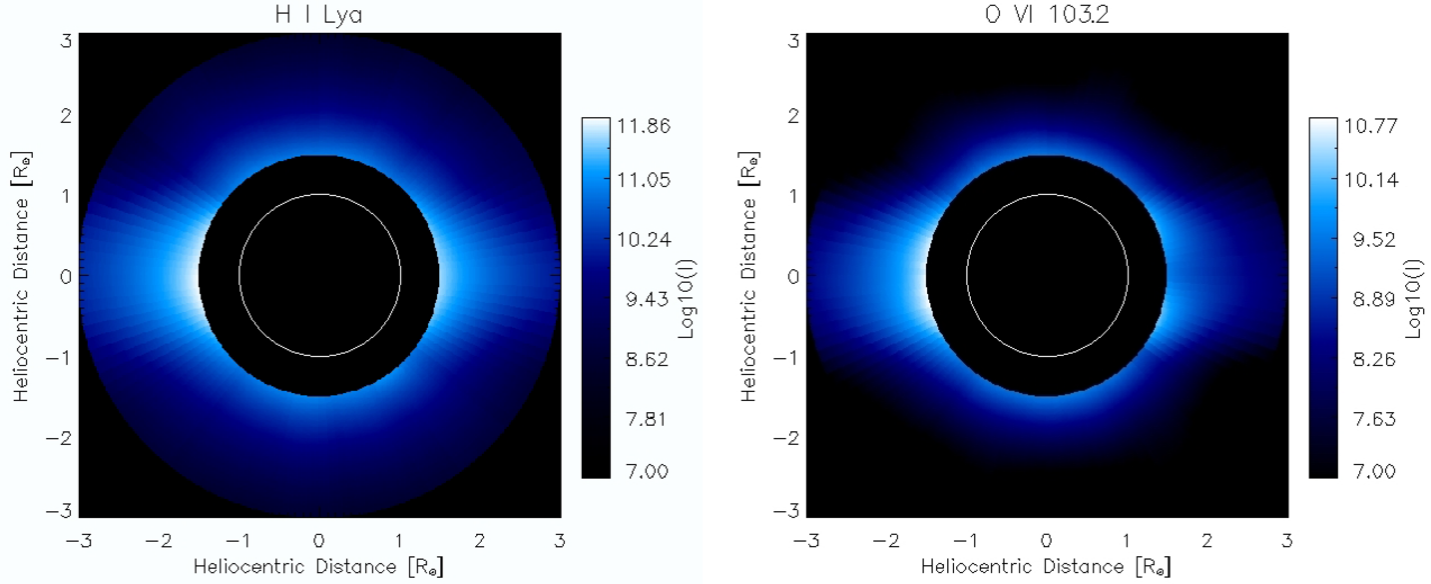
Intensity images of H i Ly$\alpha $ and O vi (103.2 nm) reconstructed from the sets of UVCS synoptic images 1 June 1996 through 3 June. Note the different morphologies above the west limb. Note that the units in the figure are given in angstroms.

Extensive UV spectroscopy of the middle corona was obtained by the *Ultraviolet Coronagraph Spectrometer* (UVCS) onboard SOHO. UVCS generally observed heights above 1.5 $\mathrm{R}_{\odot }$ (often extending out to 5 $\mathrm{R}_{\odot }$) in a wavelength range from 50.0 to 135.0 nm. Its spatial and spectral resolutions were about $7''$ and 30 km s^−1^ per pixel, but for most observations the pixels were binned due to telemetry limitations. A review is given by Kohl et al. (2006). Daily synoptic observations covered a range of heights at eight position angles around the Sun, allowing the reconstruction of intensity images, such as those shown in Figure 7.

A wide variety of plasma parameters were measured from the UVCS spectra. Line-intensity ratios among different ions of a single element yield the ionization state, which directly gives the electron temperature at low heights where the plasma is in ionization equilibrium. Once the ionization state has been established, intensity ratios of lines of different elements give their abundances; absolute abundances can be computed using the Lyman lines of H i. The column density along the line of sight is obtained from the intensity of any line, such as Ly$\alpha $, that is produced by scattering of photons from the disk. Like the densities obtained from visible-light images, these measurements yield the average density. A second approach to estimating density is to use collisionally excited lines such as Mg x, whose intensity is proportional to the density squared. In some cases density-sensitive line ratios such as O v 121.8/121.3 nm are also available.

The spectral-line widths give effective temperatures, which include both the kinetic temperatures of the ions and bulk motions due to turbulence or expansion. A unique diagnostic method using UV lines is the Doppler-dimming measurement of the velocity component away from the Sun. For example, the O vi doublet has both collisional and radiative scattering components, and their intensity ratio depends on the Doppler shift of the absorption profile away from the emission profile of disk photons. Analysis of line widths and intensities of O vi combined with Ly$\alpha $ or visible-light data makes it possible to infer temperature anisotropy. It is also possible to use Sun-grazing comets as probes to measure density, proton temperature, and wind speed at points along the trajectory, rather than integrated line of sight averages (Bemporad et al., 2007; Jones et al., 2018).

Some important results from using these methods have shown strong preferential heating of O and Mg ions compared to H in coronal holes and at heights above 3 $\mathrm{R}_{\odot }$ in streamers (Cranmer et al., 1999; Strachan et al., 2002; Frazin, Cranmer, and Kohl, 2003). Strong oxygen temperature anisotropies in the coronal-hole plasma were also indicated. Outflow speeds increase from around 20 km s^−1^ at 1.5 $\mathrm{R}_{\odot }$ to around 550 km s^−1^ at 6 $\mathrm{R}_{\odot }$ in coronal holes (Cranmer, Panasyuk, and Kohl, 2008; Raymond et al., 2018), while reaching speeds of about 100 km s^−1^ or more by 6 $\mathrm{R}_{\odot }$ in streamers (Sheeley et al., 1997; Wexler et al., 2020). Elemental abundances in streamers show a first ionization potential fractionation (FIP fractionation; see Section 4.1) similar to that seen in the slow solar wind, but the absolute abundances in streamer cores are reduced by at least a factor of three, probably by gravitational settling (Raymond et al., 1997; Feldman et al., 1998; Uzzo et al., 2006).

Ultraviolet observations of coronal mass ejections (CMEs) in the middle corona have also determined the temperatures, thicknesses, and turbulent velocities in current sheets (Ciaravella and Raymond, 2008; Bemporad, 2008), as well as the Mach numbers and electron–ion equilibration in CME shocks (Frassati, Mancuso, and Bemporad, 2020). Studies of the energy budgets of CME ejecta have shown that they continue to be heated after leaving the solar surface and that the cumulative heating is comparable to the kinetic energy (Murphy, Raymond, and Korreck, 2011; Wilson et al., 2022).

Recent technical advances have enabled great strides in UV spectroscopy of the corona utilizing a variety of launch platforms that can provide fundamental observations of coronal plasma that are inaccessible by other means (Ko et al., 2016; Strachan et al., 2017; Laming et al., 2019), including the recently launched *Ultraviolet Spectro-Coronagraph* (UVSC) *Pathfinder* instrument, which has a thirty-fold increase in sensitivity compared with UVCS and a multi-slit design to simultaneously observe two heights. Improved spatial and spectral resolution and increased spectral range are also feasible.

#### Phenomena Observed

Structures that pervade the middle corona can roughly be divided into long-lived and dynamic phenomena. The long-lived structures are generally those that make up the background coronal environment; the dynamic phenomena are more transient, often passing through the region, and they are often influenced by the background structures.

##### Long-Lived Structures

Long-lived structures are generally larger structures that persist for weeks to months – and in certain cases even years – and make up the background coronal environment. These include streamers and pseudostreamers (e.g. Pneuman and Kopp, 1971; Wang, Sheeley, and Rich, 2007), both of which are observed in the outer corona as bright radial features extending outwards. The inner- and middle-coronal magnetic topology cannot be discerned from such observations, but large-FOV EUV observations allow the magnetic topology to be traced from the inner corona out into visible-light observations (Zhukov et al., 2008). Several studies have focused on the extended streamer structures: Rachmeler et al. (2014) used SWAP with *Coronal Multichannel Polarimeter* (CoMP: Tomczyk et al., 2008) (1074.7 nm), and *Chromospheric Telescope* (ChroTel: Bethge et al., 2011) (H$\alpha $ 656.3 nm) observations to investigate the long-term evolution of a streamer–pseudostreamer structure extending into the middle corona. Guennou et al. (2016) also used SWAP data to investigate a pseudostreamer/cavity system, determining its large-scale three-dimensional structure, temperature, and density, and its associated cavity. Separately, Pasachoff et al. (2011) used ground-based eclipse observations combined with EUV observations of a streamer structure to draw comparisons between the observations in the different passbands.

Coronal fans are another example of an extended large-scale structure, observed as fan-like structures extending off the solar limb (see, e.g., Koutchmy and Nikoghossian, 2002; Morgan and Habbal, 2007). They often overlie polar crown filaments, bending over before extending outwards and tracing out the edges of boundaries between distinct topological magnetic-field regions, and they are often observed to extend far out into the heliosphere. Seaton et al. (2013a) showed fans are the single largest source of brightness at heights above 1.3 $\mathrm{R}_{\odot }$ in SWAP 17.4 nm observations, and they can persist for multiple solar rotations. Mierla et al. (2020) extended this study and showed that some fans can persist for over a year, and they can be observed extending out to at least 1.6 $\mathrm{R}_{\odot }$ in EUV observations.

##### Dynamic Phenomena

Dynamic phenomena come in many forms, unfold over minutes to days, and include all structures that pass through the middle corona, traveling both inwards and outwards (Seaton et al., 2021; Chitta et al., 2023). The most prominent and energetic structures to pass through the middle corona are CMEs (e.g. Zhang et al., 2021). CMEs come in a range of sizes (Robbrecht, Berghmans, and Van der Linden, 2009), ranging from halo CMEs to eruptions whose angular widths are barely wider than their smaller counterparts, coronal jets (e.g. Sterling et al., 2015). These structures also have a range of speeds, from a few hundred to thousands of km s^−1^ (e.g. Yashiro et al., 2004). The faster eruptions develop a shock front ahead of the ejecta front (e.g. Zhang and Dere, 2006), which in turn can produce solar energetic particles (SEPs: Reames, 1999). CME-generated shocks can also trigger transverse waves in solar helmet streamers, which have also been observed in the middle corona (Decraemer, Zhukov, and Van Doorsselaere, 2020).

Beyond their impulsive drivers, eruptions are mainly influenced by the background corona/solar wind (e.g. Schrijver et al., 2008; Mierla et al., 2013), especially in the dense inner- and middle-coronal regions. Sieyra et al. (2020) used wide-field EUV imagers to assess where CMEs can become deflected, and found deflections often occur in the inner or middle corona, during their acceleration phase. Majumdar et al. (2020) studied the deflection of CMEs and they drew comparisons to the 3D graduated cylindrical shell (GCS) model (Thernisien, Howard, and Vourlidas, 2006; Thernisien, Vourlidas, and Howard, 2009). It is reported that the velocity and width of the CMEs become constant at heights around ≈ 3 $\mathrm{R}_{\odot }$.

The evolution of eruptions through the middle corona has been studied by many authors. Many discuss the difficulties linking structure in the EUV and visible-light passbands (e.g. Byrne et al., 2014). O’Hara et al. (2019) used unique SWAP EUV (17.4 nm) off-point observations to directly trace an eruption from EUV observations (up to ≈ 2.5 $\mathrm{R}_{\odot }$) into surrounding visible-light LASCO coronagraph observations. Although the overarching kinematics could be matched, exact features were difficult to reconcile due to the emitting plasma and differences in the observing passbands.

While not observed extensively, the middle corona should also be full of MHD wave phenomena that acts on a range of timescales. Observations from the inner corona with CoMP have revealed the presence of ubiquitous propagating Alfvénic waves (Tomczyk et al., 2007; Morton, Tomczyk, and Pinto, 2015, 2016; Morton, Weberg, and McLaughlin, 2019). The waves are present along the closed loops at the base of streamers and are also seen to leave the FOV (≈ 1.3 R_⊙_) along near radially oriented structures, suggesting that they propagate directly into the middle corona. The Alfvénic fluctuations have also been long reported in the heliosphere, where they constitute an integral part of the fast-wind streams (Bruno and Carbone, 2013). The Alfvénic waves are thought to play a key role in heating the extended corona and adding momentum to the solar-wind streams (e.g. Cranmer and van Ballegooijen, 2005; Cranmer, van Ballegooijen, and Edgar, 2007; Shoda, Yokoyama, and Suzuki, 2018). While there is some suggestion of in-situ wave generation, the majority of fluctuations observed in the heliosphere are believed to originate from within the Sun, transitioning the inner and middle corona. However, it is unknown how their journey is impacted as they pass through the dynamic and structured middle corona and is unaccounted for in current wave-driven models of the corona and heliosphere.

### Radio Wavelengths

**Figure 8:**
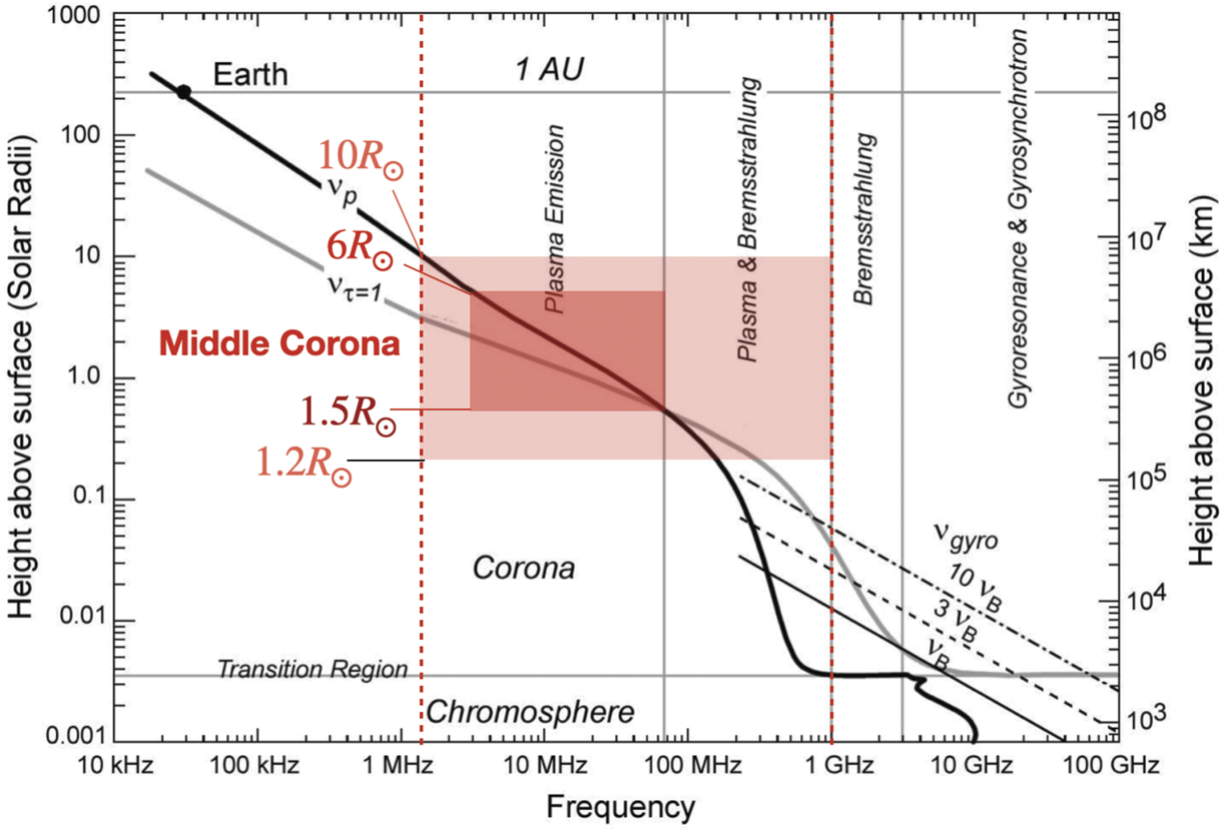
Characteristic radio frequencies in the solar atmosphere. The middle corona includes a critical region where the transition of radio-emission mechanisms occurs. The *dark-pink box* marks the nominal range of the middle corona (≈ 1.5 – 6 $\mathrm{R}_{\odot }$) and the *light-pink box* marks an extended range taking into account the highly structured and dynamic nature of the corona. The corresponding frequencies that are relevant to radio observations of the middle-corona range from < 10 MHz to ≈ 1 GHz. (Adapted from Figure 4.1 in Gary and Hurford (2004) with permission.)

From the perspective of radio observers, the middle corona includes the coronal heights where the key transition from incoherent radio emission to coherent radio emission occurs (Chen et al., 2023). Figure 8 (adapted from Gary and Hurford, 2004), shows the variation of plasma frequency ($\nu _{p}$; thick black curve), gyro-frequency ($\nu _{\mathrm{B}}$; thin black curve), and the frequency of the free–free opacity ≈ 1 ($\nu _{\tau} = 1$) layer as a function of coronal height under typical quiescent coronal conditions.

The transition region and the innermost inner corona (≲ 1.1 $\mathrm{R}_{\odot }$ from the center of the Sun) are dominated by incoherent gyromagnetic emission and free–free emission. At around 1.5 $\mathrm{R}_{\odot }$, the plasma frequency [$\nu _{p}$] layer takes over and becomes higher (closer to the observer) than both the $\nu _{\tau} = 1$ curve and the curves of $v_{\mathrm{B}}$ and its harmonics. Such a transition has a profound implication on radio observations: the quiescent free–free radio corona is no longer playing a dominant role due to the strong refraction near the plasma frequency. Meanwhile, bright coherent radio bursts, due to plasma radiation occurring near $\nu _{\mathrm{p}}$ and its second harmonic, start to be important among the observed radio phenomena. Of course, even in the region where the coherent plasma radiation dominates, incoherent radio emission from transients (e.g. CMEs) can still be observed, providing crucial diagnostics for these coronal transients, including the magnetic field and non-thermal electrons trapped in the CME or accelerated by the CME-driven shock (e.g. Bastian et al., 2001; Mondal, Oberoi, and Vourlidas, 2020; Chhabra et al., 2021). Therefore, at radio wavelengths, a broad frequency range of < 10 MHz to ≈ 300 MHz is relevant to the highly dynamic and structured middle corona (light pink box in Figure 8). It is worth emphasizing that the magnetic field and non-thermal electron distribution diagnostics in the middle corona are unique to the radio techniques, and they are otherwise difficult to achieve (if not unavailable) for remote sensing at any other wavelengths.

#### Observed Emission Mechanisms

There are numerous radio-emission mechanisms relevant to the solar corona, which include gyro-resonance (thermal electrons gyrating in the coronal magnetic field), gyro-synchrotron (non-thermal electrons gyrating in the coronal magnetic field), bremsstrahlung (or free–free; electrons interacting with ions), as well as a variety of coherent emissions such as plasma radiation (e.g. the nonlinear growth of Langmuir waves) and electron-cyclotron masers (i.e. the nonlinear growth of plasma waves at harmonics of the electron-cyclotron frequency). These emission mechanisms co-exist, but because the physical parameters differ in various coronal locations/conditions, the importance of each emission mechanism also varies. In particular, the plasma density [$n_{ \mathrm{e} }$] and magnetic field [$B$] vary dynamically throughout the corona, and hence so does the corresponding plasma frequency [$\nu _{p}$] and gyro-frequency [$\nu _{\mathrm{B}}$], thus the dominant radio-emission mechanism varies over the corona, and can change due to local conditions.

#### Radio Observations

Observing the middle corona at radio wavelengths requires a wide frequency coverage from < 10 MHz to ≈ 300 MHz (cf., Figure 8). The > 20 MHz range is generally accessible from the ground, but the lowest frequencies can only be observed from space due to the ionospheric cutoff. Currently, multiple ground-based instruments are available to observe in the frequency range relevant to the middle corona. In space, new missions, such as the *Sun Radio Interferometer Space Experiment* (SunRISE), are being designed to locate radio bursts. Figure 2 summarizes the currently operating and upcoming radio facilities that provide imaging capabilities in the frequency range relevant to middle-corona studies. This list is representative, as there are, of course, a large number of additional radio instruments that provide total-power (full-Sun integrated) dynamic spectral measurements.

Over the past decade, new advances have been made with radio facilities equipped with *broadband dynamic imaging spectroscopy*. Built on the heritage of instruments such as the *Nobeyama Radioheliograph* (Nakajima et al., 1994), *Nançay radioheliograph* (Kerdraon and Delouis, 1997), and the *Gauribidanur Radioheliograph* (GRAPH: Ramesh et al., 1998), this exciting new technique allows simultaneous imaging and spectroscopy to be performed over a broad frequency range and at a high temporal cadence. In other words, a detailed spectrum can be derived from *each pixel* in the radio image for spectral analysis. First realized by the *Karl G. Jansky Very Large Array* at the decimetric wavelengths (VLA: Chen et al., 2013) and followed by the commissioning of the *LOw-Frequency ARray* (LOFAR: van Haarlem et al., 2013), the *Murchison Widefield Array* (MWA: Tingay et al., 2013), Expanded *Owens Valley Solar Array* (EOVSA: e.g. Gary et al., 2018), and the *MingantU SpEctral Radioheliograph* (MUSER: Yan et al., 2021), this technique is just beginning to reach its full potential, using the rich diagnostics tools available (e.g. Carley et al., 2020), and it will be further explored for middle-corona sciences with the next generation of observations produced by instruments such as the *Owens Valley Radio Observatory Long Wavelength Array* (OVRA-LWA: Chhabra et al., 2021) at metric wavelengths.

#### Phenomena Observed

##### Type II Bursts and Coronal Shocks

**Figure 9:**
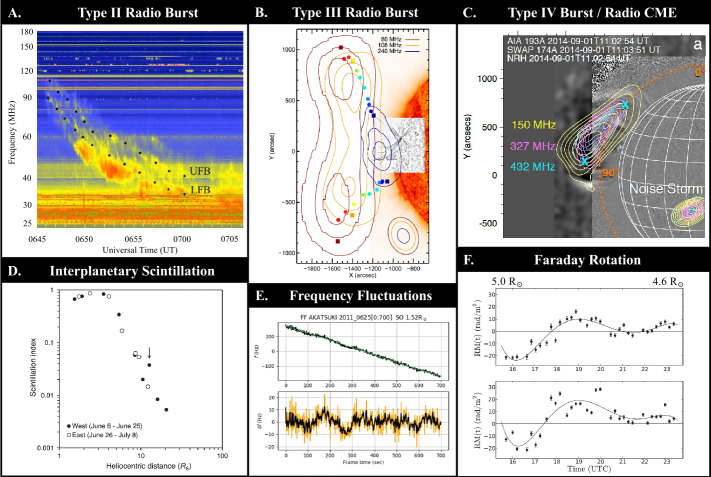
Overview of radio phenomena in the middle corona (**A**) Type-II burst with a well-defined split-band feature into an upper- and lower-frequency branch (UFB and LFB, respectively), which, if interpreted as plasma radiation from the shock upstream and downstream, can be used to estimate the shock-compression ratio and Mach number (Figure 2 in Mahrous et al. (2018), used with permission; see also Zimovets et al., 2012). (**B**) Type-III burst contours overlaid on an *Solar Dynamics Observatory* (SDO: Pesnell, Thompson, and Chamberlin, 2012)/*Atmospheric Imaging Assembly* (AIA: Lemen et al., 2012) 30.4 nm, image. Tracking the radio burst over several frequencies illustrates an evolution from a single source in the inner corona to two separate sources split between two separate flux tubes in the middle corona (Figure 14 in McCauley et al., 2017, used with permission). (**C**) Type-IV burst associated with a radio CME resulting from trapped non-thermal electrons emitting gyrosynchrotron radiation, which can be used to determine the CME’s magnetic-field strength (Figure 2 in Carley et al., 2017, used with permission). (**D**) The scintillation index (representing the magnitude of the intensity fluctuations) as a function of heliocentric distance; intensity scintillation provides information on the plasma density and solar-wind speed (Figure 3 in Imamura et al., 2014, used with permission). (**E**) Frequency fluctuations provide information on plasma-density fluctuations and solar-wind speed. *Upper panel* shows raw frequency data dominated by a Doppler shift and the *bottom panel* shows the frequency fluctuations with the Doppler shift removed (Figure 2 in Wexler et al., 2020, used with permission). **(F**) Faraday rotation provides information on the plasma density and magnetic-field component along the line of sight. Differences between measurements along two closely spaced lines of sight (provided here by a background radio galaxy) can be used to probe coronal electric currents (Figure 5 in Kooi et al., 2014, used with permission).

Type-II radio bursts are seen from metric to kilometric wavelengths (a few times 100 MHz to tens of kHz) and are notable for their relatively slow drift to lower frequencies, compared to Type-III radio bursts (see, e.g., the empirical expression of their drift rate by Aguilar-Rodriguez et al., 2005). They result from coherent plasma radiation of energetic electrons accelerated at or near the shock front, propagating outwards at super-Alfvénic speeds. Therefore, they provide important diagnostics for both the shock parameters and shock-accelerated electrons. Figure 9A shows an example of a metric Type-II burst that shows a split-band feature in the time–frequency domain. This feature is interpreted as plasma radiation at the shock upstream and downstream regions, which in turn can be used to estimate the shock-compression ratio and Mach number. Recently, thanks to the imaging spectroscopy capability provided by instruments such as LOFAR, new insights have been provided into their source region at the CME-driven shock front. For example, Morosan et al. (2019) found shock-accelerated electrons “beaming out” from multiple acceleration sites located at the nose and flank of the shock.

##### Type-III Bursts and Electron Beams

Type-III radio bursts are produced by fast electron beams (≈ 0.1 – 0.5 c) escaping along open magnetic-field lines (see, e.g., Reid and Ratcliffe, 2014; Reid, 2020 for recent reviews). Observations of Type-III bursts span an extremely wide frequency range from > GHz to kHz and exhibit a much greater frequency drift than that of Type-II bursts.

In the middle corona, these bursts are predominantly associated with open-field lines. With imaging spectroscopy provided by general purpose facilities such as LOFAR and MWA, new advances have been made in tracing the trajectories of the electron beams (e.g. Panel B in Figure 9), which in turn outline the electron-beam-conducting magnetic-field lines in the middle corona (e.g. McCauley et al., 2017; Mann et al., 2018). The emission frequencies and fine structures in the dynamic spectra have been used to derive the coronal density variation in height and properties of the coronal turbulence (Kontar et al., 2017; McCauley, Cairns, and Morgan, 2018; Mann et al., 2018; Reid and Kontar, 2021).

##### Type-IV Bursts and Trapped Electrons

Type-IV radio bursts are broadband bursts characterized by their slow- or non-drifting appearance in the radio dynamic spectrum. Typically observed after the flare peak, they are thought to be produced by non-thermal electrons trapped in closed coronal structures, emitting coherent (plasma or electron-cyclotron maser) radiation and in some cases incoherent gyrosynchrotron radiation. Depending on the underlying emission mechanism, Type-IV bursts can, on one hand, trace and outline the closed magnetic structure of interest, and, on the other hand, provide diagnostics of the source region (see, e.g., review by Carley, Vilmer, and Vourlidas, 2020, and references therein). First detected and named in the 1950s (Boischot, 1957), Type-IV radio bursts have been generally sub-categorized into stationary and moving Type-IV bursts. The latter, by virtue of their close association with CMEs, are of particular interest because of their diagnostic potential for CME magnetic fields and energetic electrons.

##### Radio CMEs

Faint radio emissions that closely resemble their visible-light CME counterparts are dubbed “radio” CMEs because of their similar appearance (e.g. Panel C in Figure 9). In fact, they were discovered around the same time as LASCO’s start of science operations in 1996 (see recent review by Vourlidas, Carley, and Vilmer, 2020). Since the emission occurs at large harmonics of the electron gyrofrequency, this emission can be found at frequencies above the local plasma frequency, thereby being less affected by scattering effects. Thanks to their incoherent nature, when imaged at multiple frequencies, they can be used to map the coronal magnetic field and non-thermal electron distribution associated with the CMEs (see, e.g., Bastian et al., 2001; Maia et al., 2007; Mondal, Oberoi, and Vourlidas, 2020).

##### Propagation Effects

The propagation effects of radio waves provide other means for studying the middle corona. These observations utilize a known, point-like background radio source (e.g. a spacecraft transmitter or a natural celestial source such as a pulsar or radio galaxy) to “shine through” the corona. The observed radio signatures can be used to probe the structure and dynamics of the middle corona. Importantly, these trans-coronal radio-sensing methods are applicable in all solar-activity states and do not rely on observations of specific episodic outburst phenomena. Signal delays at different frequencies (i.e. dispersion measure) can be used to constrain the coronal density. Signal broadening and scintillation provide information on the density inhomogeneities in the turbulent coronal plasma (Rickett, 1990). Analysis of radio scintillation and frequency fluctuations (Panels D and E in Figure 9) can provide estimates of solar-wind speed (Imamura et al., 2014; Wexler et al., 2019, 2020). In addition, modulations of the signal polarization due to Faraday rotation (Panel F in Figure 9) can be used to constrain the coronal magnetic field and its fluctuations (see, e.g., Wexler et al., 2017; Wexler, Jensen, and Heiles, 2021; Kooi et al., 2022, and references therein).

## Properties and Transitions in the Middle Corona

The inner corona exhibits a broad range of temperatures, which can exceed $T>10^{7}~\text{K}$ in the case of flares, and electron densities of $n_{ \mathrm{e} }\approx 10^{15}\text{ m}^{-3}$ in closed structure regions. These closed magnetic-field regions are generally associated with the relative confinement of plasma, with subsonic flow speeds, and increased elemental abundances. The open-field configurations associated with coronal holes are known to produce fast solar winds and relatively low scale-heights, and they exhibit FIP elemental abundances close to those of the photosphere. In contrast to the inner corona, the outer corona is generally a region of supersonic solar-wind outflow, with an open magnetic-field pattern and stabilized ionization-charge states.

The characteristics of the middle corona straddle those of the inner and outer corona, and accordingly the region hosts a number of structural, dynamic, and plasma-physics transitions, as described in Table 1. The most important structural change is the transition from a mix of open and closed magnetic configurations to almost exclusively open-field structures.

Due to instrumental limitations, the middle corona has not been continuously or comprehensively probed by instruments that can provide self-consistent plasma parameters. As a result, the multiple physical transitions that occur here have not been fully characterized, and methods to study plasma properties must include extrapolation and modeling, often drawn from measurements of surrounding regions (e.g. Lynch, 2020; Schlenker et al., 2021). Table 2 presents a list of canonical plasma properties measured/derived on either side of the middle corona illustrating the transitions that occur within the region.

**Table 2:** Representative middle-corona properties in fast and slow solar-wind regions. The *top portion* includes representative measured and modeled quantities, the *bottom portion* includes derived quantities.

Symbol	1.5 $\mathrm{R}_{\odot }$		6.0 $\mathrm{R}_{\odot }$		Units: Definition
	Fast	Slow	Fast	Slow	
${n_{\mathrm{e}}}$^a^	1 × 10^12^	7 × 10^12^	6 × 10^9^	3 × 10^10^	$\mathrm{m}^{-3}$: electron no. density
$T_{\mathrm{p},\parallel}$^b^	1.6	2.0	1.9	0.85	MK: proton ∥ temperature
$T_{\mathrm{p},\perp}$^b^	2.0	2.6	—	1.1	MK: proton ⊥ temperature
$T_{\mathrm{e}}$^c^	1.4	1.8	0.8	—	MK: electron temperature
$T_{\mathrm{O},\parallel}$^d^	2	> 1	60	> 5	MK: oxygen ∥ temperature
$T_{\mathrm{O},\perp}$^d^	10	20	200	20	MK: oxygen ⊥ temperature
$V_{\mathrm{SW}}$^e^	> 100	< 25	550	150	km $\mathrm{s}^{-1}$: outflow speed
He/H^f^	—	8%	—	—	— : helium/hydrogen ratio
${\mathrm{FIP}_{\mathrm{bias}}}$^g^	1.5 – 2.5	4 – 6	—	—	— : elemental composition compared to photospheric composition
*B*^h^	1.3 ×10^5^	7 × 10^4^	4 × 10^3^	4 × 10^3^	nT: magnetic field
$C_{\mathrm{S}}$	150	170	160	100	km s^−1^: sound speed
$V_{\mathrm{A}}$^i^	3000	600	1100	500	km s^−1^: Alfvén speed
$\omega _{\mathrm{pe}}$	5.6 × 10^7^	1.5 × 10^8^	4.4 × 10^6^	9.8 × 10^6^	Hz: $\mathrm{e}^{-}$ plasma frequency
*β*^j^	< 0.01	≥ 0.08	< 0.1	≥ 0.04	plasma-*β*, $P_{\mathrm{gas}}/P_{\mathrm{mag}}$

^a^ Bird and Edenhofer (1990), Guhathakurta et al. (1999), Raymond et al. (2018), Wexler et al. (2019).

^b^ Strachan et al. (2002), Frazin, Cranmer, and Kohl (2003), Cranmer, Panasyuk, and Kohl (2008), Cranmer (2020).

^c^ Raymond et al. (1997), Cranmer et al. (2009).

^d^ Strachan et al. (2002), Frazin, Cranmer, and Kohl (2003), Cranmer, Panasyuk, and Kohl (2008).

^e^ Woo (1978), Strachan et al. (1993), Raymond et al. (2018), Wexler et al. (2020), Romoli et al. (2021).

^f^ Moses et al. (2020).

^g^ Feldman et al. (1998), Young, Klimchuk, and Mason (1999), Raymond et al. (1997), Uzzo, Ko, and Raymond (2004).

^h^ Kooi et al. (2022), Yang et al. (2020), Wexler, Jensen, and Heiles (2021), Alissandrakis and Gary (2021), Hofmeister et al. (2017).

^i^ Evans et al. (2008).

^j^ Gary (2001) for slow SW; note $\beta =\frac{C_{\mathrm{s}}^{2}}{V_{\mathrm{A}}^{2}}$.

Note: 1 gauss (cgs) = 10^5^ nT = 10^−4^ T (mks, S.I.).

The parameters in Table 2 are also subdivided into categories of fast and slow solar wind to represent the range of values that are present in the different regions. Coronal holes are considered the source of the fast solar wind, streamers, and pseudostreamers contain slow solar wind, and the remaining regions are predominantly slow, interspersed with some fast regions. In slow-solar-wind and streamer regions, the supersonic solar-wind outflow is achieved by approximately 5 – 6 $\mathrm{R}_{ \odot }$ (Sheeley et al., 1997; Wexler et al., 2020).

As discussed in Sections 3.1 and 3.2, several instruments, including UVCS and various radio arrays, have sporadically provided direct diagnostics of specific middle-corona properties, yielding estimates of density, proton temperature, ion temperatures, temperature anisotropy, outflow speed, ionization state, and elemental composition. However, even for the relatively simple case of a quiet-Sun streamer, various different estimates of the densities and temperatures have been published (Del Zanna et al., 2018; Seaton et al., 2021). This might be due to the large-amplitude density contrasts on small scales (Raymond et al., 2014) and estimates based on scattered light (average density) or emission lines (average density squared).

To a good approximation, the magnetic field in the inner corona is force-free since the plasma-$\beta $ is much smaller than unity. Throughout the middle corona, the magnetic control is only partial. The confinement of plasma by closed fields diminishes. At the same time, the stabilization or “freeze-in” of the ionization-charge states occurs. This provides the basis for source region diagnostics based on measurements far from the middle corona. From the global heliospheric magnetic-field modeling point of view, the middle corona is critical; the PFSS is nominally placed between 2.5 and 3.0 $\mathrm{R}_{\odot }$, but actually may be more suitably placed at different middle-corona region altitudes (Lee et al., 2011). With these several key transitions occurring over a relatively small radial distance range, intensive cross-disciplinary analysis is necessary to create internally consistent models of the complex processes.

### Elemental Composition

It is now well-established that the chemical composition of the corona varies depending on the structures observed, and it differs from the solar photospheric composition, although both recent revisions of older data and new analyses indicate that, at least up to 1 MK, the composition of the quiet solar corona is close to photospheric (Del Zanna and Mason, 2018; Del Zanna et al., 2018; Madsen et al., 2019). The variability in chemical abundances depends, among other factors, on the first ionization potential (FIP) of the element and gravitational-settling effects.

The FIP effect is a process in which elements with neutral atoms with ionization potentials below 10 eV (e.g. Fe, Si) are preferentially enhanced by a factor of two to four relative to those with higher FIP values (e.g. O, Ne). The FIP effect is most prominent in active regions and helmet streamers at the Sun and is also reflected in the in-situ observations of the slow solar wind and SEPs that originate from those structures (Geiss, Gloeckler, and von Steiger, 1995; von Steiger et al., 2000; Uzzo et al., 2006; Baker et al., 2013; Reames, 1999). In coronal holes and the fast wind, the FIP enhancement is small or non-existent (Feldman and Widing, 1993).

Gravitational stratification (settling) of higher-mass elements (compared to lighter ones) can appear in large, long-lived coronal structures, such as the cores of helmet streamers, which are observed throughout the middle corona (Raymond et al., 1997). Spectral observations of helmet streamers from UVCS have shown a significant depletion of both low (FIP < 10 eV) and high (FIP > 10 eV) FIP elements (O, Si, Mg) in accordance to particle mass that is thought to be caused by gravitational settling taking place high in the corona (Uzzo et al., 2003; Uzzo, Ko, and Raymond, 2004).

A similar effect was observed by the *Solar Ultraviolet Measurements of Emitted Radiation* (SUMER: Wilhelm et al., 1995) instrument on SOHO. This phenomenon results in mass fractionated coronal plasma where the loop apex becomes depleted of the heaviest elements as they sink towards the footpoints faster compared to lighter elements.

The gravitational settling shows strong spatial dependence, such that it becomes less pronounced between the helmet-streamer core and legs. This variation is attributed to the transition between closed (core) and open/closed field, (streamer edge) where plasma confined to the streamer core resides in the corona long enough for notable gravitational settling to take place, ≈ one day, while plasma on the open/closed field boundary is released on a faster timescale (Lenz, Lou, and Rosner, 1998). These observations indicate that gravitational settling can be important in regulating the plasma’s chemical composition in large coronal loops, and can be a distinctive compositional signature of helmet-streamer plasma observed in the form of heavy-element dropouts in the solar wind and CMEs at 1 AU (Weberg, Zurbuchen, and Lepri, 2012; Weberg, Lepri, and Zurbuchen, 2015; Rivera et al., 2022b). These are believed to correspond to pulses of gas released from the cusps of helmet streamers by magnetic reconnection. However, further examination of gravitational settling and variability in the chemical composition across the middle corona is necessary to further characterize solar-wind origin and the pathways to its formation and connection to heliospheric structures.

### Charge State Evolution and Freeze-in Distances

One important transition point occurring in the middle corona is the height at which heavy-ion abundances in the solar wind and CMEs reach their “freeze-in” altitude (Hundhausen, Gilbert, and Bame, 1968; Owocki, Holzer, and Hundhausen, 1983). The freeze-in process takes place as charge states become fixed at some radial distance from the Sun, where the plasma becomes too tenuous to sustain collisional ionization and recombination processes any further. After this transition, ions become uncoupled from thermodynamic changes in the plasma and remain fixed throughout the solar wind’s radial evolution.

In-situ measurements of ions can be tied to their sources in the corona by comparing frozen-in populations. As a result, freeze-in states can be used to probe the heating and cooling in the nascent solar wind prior to freeze-in.

**Figure 10:**
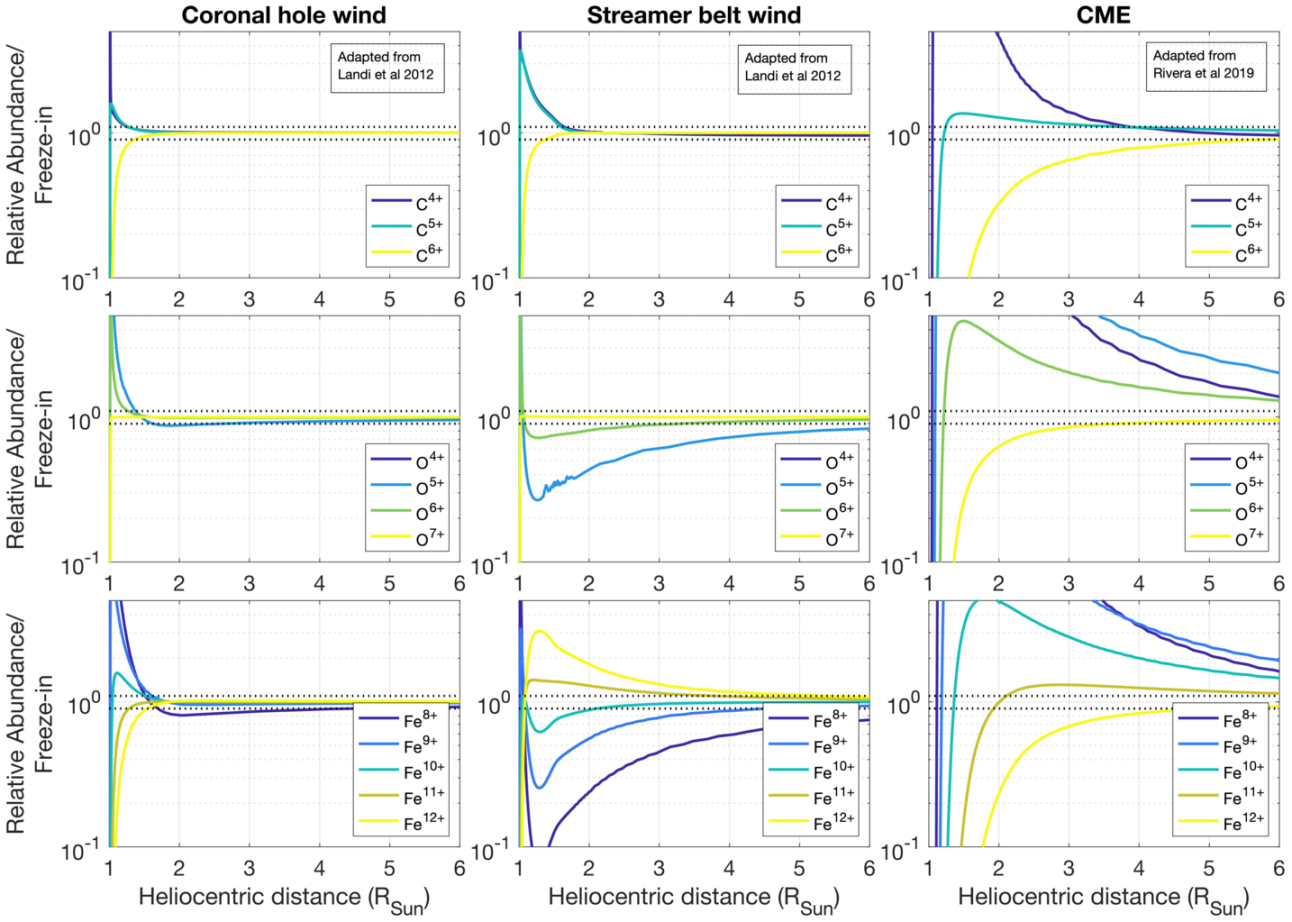
Radial evolution for selected C, O, Fe ions within simulated coronal-hole wind, equatorial streamer-belt solar wind, adapted from Landi et al. (2012) and a CME adapted from Rivera et al. (2019). The *horizontal dashed lines* represent ions reaching 10% of their freeze-in value.

Freeze-in distances are governed by the plasma’s electron temperature, density, and outflow speed, which result in large ranges of freeze-in distances among solar structures throughout the middle corona. Boe et al. (2018) used the regions where the resonant scattering dominates in visible-light observations of Fe^10,13+^ as a proxy to estimate freeze-in distances. They found in coronal holes and helmet-streamers distances of 1.25 – 2 $\mathrm{R}_{\odot }$ and 1.45 – 2.2 $\mathrm{R}_{\odot }$, respectively. However, theoretical freeze-in heights can be considerably larger in pseudostreamers (Shen et al., 2018). Similarly, simulations of the solar wind have shown that freeze-in distances for other ions of C, O, and Fe in coronal-hole wind can range between 1 – 2 $\mathrm{R}_{\odot }$, while in equatorial-streamer-belt wind ions may evolve beyond 5 $\mathrm{R}_{\odot }$, as shown in the left and middle columns of Figure 10 (Ko et al., 1997; Landi et al., 2012; Gilly and Cranmer, 2020).

In CME plasma, ion freeze-in distances are predicted to reach beyond 6 $\mathrm{R}_{\odot }$ in the dense prominence core, as shown in the right column of Figure 10 (Rivera et al., 2019). Also, the higher velocities in CMEs can be an important factor in the higher freeze-in heights (Rakowski, Laming, and Lepri, 2007). Simulations of the freeze-in process using non-equilbrium ionzation (NEI) conditions have enabled studies of the corona’s thermodynamic state using heavy-ion composition that reflect the plasma’s early stages of ionization evolution (see, e.g., Landi et al., 2012; Gilly and Cranmer, 2020). Multi-wavelength observations throughout the middle corona will place more stringent constraints on the ion evolution at these critical freeze-in heights to strengthen the connection made between the Sun and in-situ observations made by spacecraft.

Anomalous charge states observed at 1 AU are believed to arise in the middle corona, where CMEs show very high (Fe^+17^) and very low (Fe^+3,+4^) charge states. Anomalous dropouts of fully stripped ions such as C^+6^ are also observed. However, there is no clear explanation of how these bare-ion dropouts occur (Kocher et al., 2017; Zhao et al., 2017; Rivera et al., 2021).

### Magnetic Topology of the Middle Corona

There are three types of large-scale features that dominate the middle corona, each associated with a distinct magnetic topology: the closed streamers, including their cusps, rays of various types, and open-field regions. The three have unique characteristic speeds, densities, plasma-$\beta $, composition, and FIP values. (An additional, transient, topological feature is large-scale closed-loop systems, or giant arches, formed by magnetic reconnection during large eruptions, which can reach well into the middle corona and persist in active regions for days to weeks; West and Seaton, 2015.)

Coronal holes undergo several changes in the middle-corona region: the magnetic field expands super-radially and fast solar-wind acceleration occurs, generally in the lower reaches of the middle corona (Cranmer, 2009, and references therein). The cause of this acceleration is still a topic of some debate, and is one of the middle corona’s most important open questions. In contrast, the slow solar wind is organized in the middle corona and initial acceleration to the supersonic threshold occurs. The wind is believed to become super-Alfvénic at varying distances between 10 – 25 $\mathrm{R}_{\odot }$ into the extended corona and heliosphere (Wexler et al., 2021); where the kinetic energy dominates over the magnetic energy, regardless of the value of the plasma-$\beta $. The middle corona is important for mediating the overall morphology of coronal-holes: the high rate of forced reconnection in the “magnetic carpet” of the solar photosphere (Simon, Title, and Weiss, 2001) induces a high diffusion rate of small-scale magnetic flux (Hagenaar et al., 1999), which should break up large-scale coronal holes on a time scale of days; this is not observed (Cranmer et al., 2009), implying that the structure of the open flux is somehow communicated downward from or through the middle corona, to affect reconnection patterns near the surface.

Helmet-streamer cusps lie in the middle-corona region; these form the heliospheric current sheet, as well as some secondary topological surfaces (e.g. above polar crown filaments: Rachmeler et al., 2014). Here, high-$\beta $ plasma and magnetic-field fluctuations near the magnetic $y$-points pinch off to form plasmoids or “blobs” (Wang and Hess, 2018). While helmet streamers are generally fairly quiescent and contain the only magnetic field not directly connected to the solar wind in this region, they are also the source of streamer blow-outs, some of the largest and most internally coherent CMEs in the heliosphere (Lynch et al., 2010; Vourlidas and Webb, 2018).

Rays are a term that can be applied to any of various structures of the same basic null-point topology. Plumes and jets, which generally lie in open-field regions, have extensive collimated columns of enhanced-density plasma extending above their domes in the inner corona, which have been observed to extend into the heliosphere, with direct imaging as high as 40 $\mathrm{R}_{\odot }$ (DeForest et al., 1997; Del Zanna, Bromage, and Mason, 2003; Raouafi et al., 2016b; Karpen et al., 2017; Kumar et al., 2019; Uritsky et al., 2021). They have long been postulated as a small but relatively stable source of contributions to the solar wind, and some middle-corona observations show outflows into such smaller-scale features (Seaton et al., 2021). Recent observations have revealed quasi-periodic energy releases and jetlets (period = five minutes) at the base of plumes, which are important to understand the coronal heating and origin of solar wind in plumes (Kumar et al., 2022).

Pseudostreamers, which are similar to streamers in appearance, but topologically more complex, also have outer spines that are often seen in coronagraph imagery, potentially appearing as miniature low-lying streamer cusps, as the narrow spines themselves, or rarely as dim fans curving away from the dome surface, depending upon the height and angle of viewing. Evidence shows that coherent magnetic structures attempting to escape the inner corona can be destroyed by reconnection in these null-point topologies, leading to large streams of unstructured plasma being ejected into the solar wind from these narrow rays (Vourlidas et al., 2017; Kumar et al., 2021; Mason, Antiochos, and Vourlidas, 2021; Wyper et al., 2021). In addition, pseudostreamers can produce unstructured, slow CMEs (Wang, 2015) as well as “bubble-shaped” fast CME (> 1000 km s^−1^) via interchange/breakout reconnection at 3D null-points (Kumar et al., 2021).

This collection of qualities is described by the S-web, a map of separatrices and quasi-separatrix layers (QSLs) in the heliosphere (e.g. Antiochos et al., 2011). The major separatrix lines show the Heliospheric Current Sheet (HCS), while the quasi-separatrix layers are smaller arcs corresponding to pseudostreamers, jets, etc. Taken together, the topological picture of this region is diverse and rich; the closed but dynamic streamer belt regularly extrudes blobs of closed field and relatively dense plasma into the otherwise narrow and well-structured heliospheric current sheet. Much of the remaining volume is filled in by the expanding field and tenuous plasma of the coronal-holes, occasionally punctuated by tight spears of condensed field and plasma introduced by null-point topologies. New large-FOV EUV observations have recently provided direct imaging of the S-web and its complex dynamic behavior in the middle corona (Chitta et al., 2023), validating models that predicted its importance in governing the topological and dynamic transitions that occur here.

## Modeling the Middle Corona

Because of the multiple physical transitions within the middle corona – and the instrumental limitations that have hampered a complete characterization of them – a unified model of middle-corona physics does not yet exist. The lack of continuous, comprehensive measurements of the region as a whole has also limited the availability of high-quality, data-based model boundary and initial condition parameters. However, a limited number of direct measurements from UVCS and various radio arrays have provided estimates of density, proton temperature, ion temperatures, temperature anisotropy, outflow speed, ionization state, and elemental composition. (See Section 4 for a thorough discussion.)

### Spectral Diagnostics and Implications for Forward Modeling

A general description of the underlying atomic data needed to model the coronal emission and obtain information about the plasma state appears in the *Living Review* by Del Zanna and Mason (2018). Modeling the visible/IR continuum emission resulting from the solar-disk radiation being Thomson-scattered by the free electrons is relatively simple, although a knowledge of the spatial distribution of the electron density is required. Following van de Hulst (1950), in most cases the modeling assumes a homogeneous distribution with spherical/cylindrical symmetry. This is routinely used to infer the radial-density profile from measurements of the polarized Brightness (pB). However, this is an over-simplification, as the corona is known to be finely structured (e.g. the images by Habbal, Morgan, and Druckmüller, 2014, and many other similar solar-eclipse observations). More sophisticated approaches can provide density diagnostics using broadband visible-light imaging without the simplifying assumptions of homogeneous distributions and spherical symmetry (as in the van de Hulst inversion), for example Decraemer, Zhukov, and Van Doorsselaere (2019), provided diagnostics using a more elaborate geometric approach.

As the middle quiescent corona appears to have an electron temperature around 1 MK (Boe et al., 2020; Seaton et al., 2021), it emits a range of coronal lines from the X-rays (above 2.0 nm) to the near infrared, mostly from highly ionized atoms. The strongest coronal lines are allowed transitions in the EUV/UV, between 17.0 and 110.0 nm, and forbidden transitions in the visible and near-infrared. The modeling of most of the allowed transitions is relatively simple, as their emissivity mainly depends on the local electron density and temperature, as well as chemical abundances and ionization states. The main populating mechanism is excitation by electron collisions (collisional excitation), and the observed radiance is proportional to the square of the electron density. However, there are cases where photo-excitation by the solar-disk radiation in the visible/near-infrared affects the ion populations, as in the case of Fe xiii (see, e.g., Dudík et al., 2021), which somewhat changes the predicted emission of the allowed transitions. Measurements of the density from line ratios are available for the inner corona.

There is a range of allowed transitions from neutrals or ionized atoms that become very strong by direct resonant photo-excitation from the solar-disk radiation. Examples are lines from H i, He i, He ii, and O vi, which are the strongest in the inner and middle corona. Such atoms produce a very strong solar-disk emission from the chromosphere/transition region, and naturally produce little emission at coronal temperatures/densities. Hence, a large fraction of their coronal emission is produced by resonant photo-excitation. Their modeling is complex as it depends strongly on the distribution of the solar-disk radiation, which is highly variable and, in the case of He, also controls the charge states via photo-ionization. Modeling the helium emission has several extra complications, as illustrated by the first of the coronal models of Del Zanna et al. (2020). Lines from these ions offer several diagnostics, the most widely used one being Doppler dimming, to measure the outflow velocity (see, e.g., Noci, Kohl, and Withbroe, 1987).

**Figure 11:**
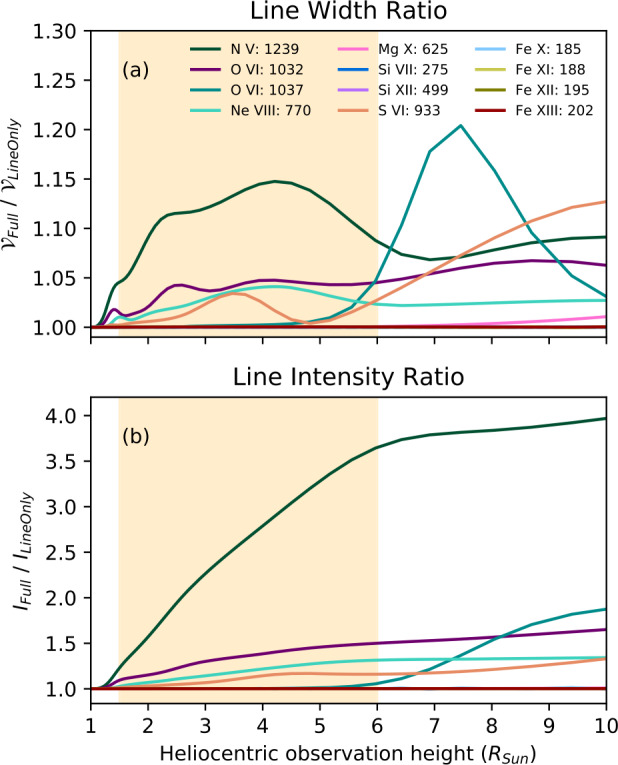
Ratio of modeled ion-line properties as a function of height, with and without including a modeled continuum in the resonantly scattered light. Panel **a** indicates excess line-width caused by including the continuum, while Panel **b** shows excess intensity. The *shaded orange area* indicates the middle-coronal region. Ion-line wavelengths are given in units of angstroms. Adapted from Figure 14 of Gilly and Cranmer (2020), and used with permission.

Doppler effects must be considered when forward modeling the corona, or the calculation will be incomplete. Figure 11 shows the difference between two runs of the Global Heliospheric Optically thin Spectral Transport Simulation (GHOSTS) code (Gilly and Cranmer, 2020), which simulates both collisional excitation and resonant scattering along lines of sight over the North Solar Pole. The only change between the two runs is the choice of incident-light profile in the resonant-scattering calculation: The “Full” case uses a window that contains realistic continuum out to the edge of the Doppler-shifted scattering window, while the “Line Only” case simply uses a model Gaussian spectral line. Figure 11a shows the change in the full width at half maximum of the lines produced in each case, which can be as much as 15% in the middle corona. The effect on line intensities is much greater: Figure 11b demonstrates that some lines can be brightened by a factor of 1.5 – 4. Because of these effects, it is important to include a sufficient range into and out of the plane-of-the-sky along the line of sight, and the incident-light profile used in the scattering calculations must be wide enough and include a realistic continuum component such that the scattered light profile does not artificially truncate.

Calculations of the collisional-excitation rates, which started in the 1970s, have now reached, for a few key ions, an accuracy of the order of 10 – 20%. (For a recent review of a series of calculations for astrophysical ions, see Badnell et al., 2016). Calculations of the decay rates for spontaneous emission now have an even better accuracy (see, e.g., the review by Jönsson et al., 2017). The atomic rates for the coronal ions in the EUV/UV are relatively complete and accurate, as a series of benchmark studies has shown (see Del Zanna, 2020, 2019; Del Zanna and Mason, 2018, and references therein), although significant improvements in the soft X-rays are still needed (Del Zanna, 2012). The latest set of atomic rates made available to the community is included in CHIANTI version 10 (Del Zanna et al., 2021). The database also includes some approximate treatment of resonant scattering.

The ionization state is controlled by collisions with free electrons. The ionization and recombination rates, which are needed to calculate the ion abundances, either in equilibrium or not, are somewhat more uncertain. Fortunately, the modeling of the ionization state is relatively simple as most of the ion populations are in their ground state, hence it only depends on the electron temperature. Note, however, that there can be cases when photo-ionization from the solar disk can affect the ion balance in the corona and the solar wind (Landi and Lepri, 2015).

In summary, to model the radiances of the allowed transitions not affected by resonant photo-excitation, knowledge of the electron density and temperature is needed. Estimates of the averaged density in the middle corona are widely available via the pB-measurements and the van de Hulst (1950) inversion. However, direct measurements of the electron temperature have been lacking (Del Zanna and Mason, 2018). This is one of the major problems when modeling the middle corona.

Therefore, modeling usually relies on the temperature obtained from line ratios assuming that ionization equilibrium holds, i.e. the ionization temperature. That is usually a reasonable assumption in the low quiescent corona, but not necessarily in the middle corona, where the ionization state needs to be calculated taking into account estimates of local flows, densities, and temperatures.

Strong emission in the middle corona is also produced by forbidden lines in the visible/near-infrared by highly ionized atoms; see the review by Del Zanna and DeLuca (2018). There are also many weaker forbidden lines in the UV. As in the case of the allowed transitions by neutral or low-charge ions, these lines are photo-pumped by the solar-disk radiation. The advantage of such transitions is that they are visible out to great distances (cf. Habbal et al., 2011). However, they are also complex to model and use for diagnostic purposes.

Accurate collisional-excitation rates for these forbidden lines are difficult to obtain as they require large-scale scattering calculations. Such calculations for iron ions have shown significant increases (50 – 100%) in the predicted emissivities of some key transitions (cf references in Del Zanna and Mason, 2018). However, not all ions have accurate atomic rates available. Also, large-scale models that are not currently available in CHIANTI are needed to account for all the cascading effects from high-lying states.

For any atom affected by resonant photo-excitation, accurate estimates of the solar-disk radiance are needed. For the visible and near-infrared lines, this is achievable, as the solar-disk radiance has little variability, but it is more challenging for UV lines, because radiance from the solar disk and inner corona is both variable and inhomogeneous (Vernazza and Reeves, 1978). Also, an accurate knowledge of the local density is needed to calculate the relative contribution of the collisional-excitation and resonant photo-excitation processes. Obtaining densities from, e.g., line ratios of forbidden lines is not trivial: The plane-of-the-sky approximation is reasonable for the allowed transitions not affected by resonant photo-excitation, but the lines affected have a significant long-range contribution (see, e.g., Yang et al., 2020; Del Zanna et al., 2023). As a consequence, measurements of the ionization temperature from the resonant photo-excitation lines becomes strongly dependent on the distributions of the electron densities. The same issues apply when measuring chemical abundances.

Detailed knowledge of the emission mechanisms that act in the middle corona has led to the development of a variety of forward models and modeling frameworks (e.g. FORWARD: Gibson et al., 2016). However, as knowledge of the nature of emission from the middle corona is quickly evolving, the terrain for modeling this region is also shifting rapidly. Such forward models have been used both to characterize middle-corona structure and improve understanding of the emission sources themselves, which is critical for developing more robust plasma diagnostics.

**Figure 12:**
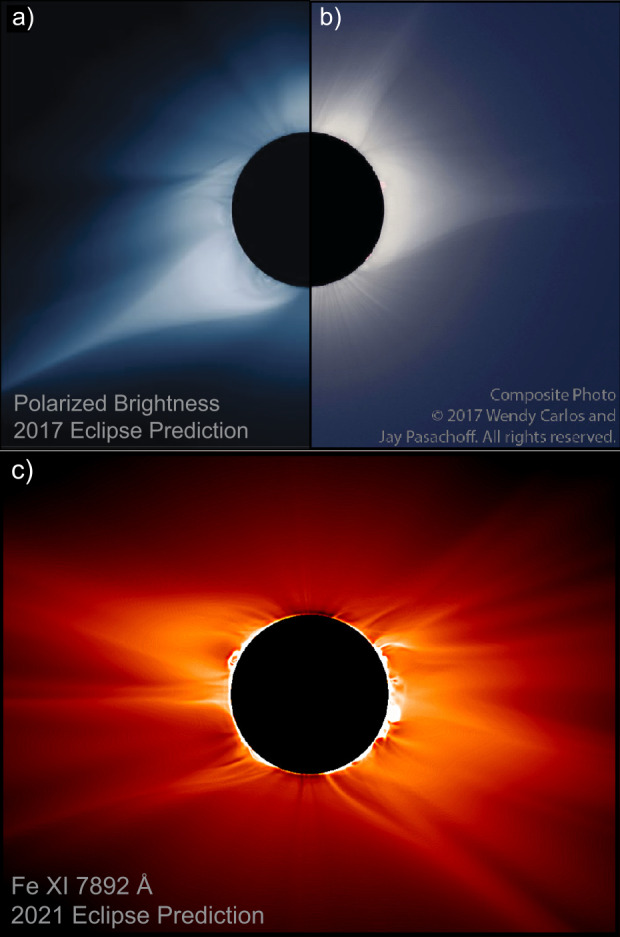
Two examples of forward modeling from the PSI eclipse predictions. *Top panels*: a merged image comparing the polarized brightness prediction for the 21 August 2017 total solar eclipse (**a**) to a processed eclipse photo (**b**, images adapted from Mikić et al., 2018, and used with permission). Panel **c** shows radially filtered, sharpened radiances for the photoexcited Fe xi 789.2 nm emission line for the 14 December 2021 eclipse prediction (predsci.com/eclipse2021, see Boe et al., 2022, for details on the method).

The best-known examples of forward models that include the middle corona are probably the Predictive Science Inc. eclipse predictions, which capture global coronal structure extending out through the middle corona, in an attempt to predict the corona’s appearance across a variety of wavelengths, prior to a total eclipse (www.predsci.com/corona, see Mikić et al., 2018, for details on the method). These predictions leverage the Magnetohydrodynamic Algorithm outside a Sphere (MAS; see additional discussion in Section 5.3) global coronal model, which has also been used to extensively characterize the topology and thermodynamics of the corona, addressing a number of open questions about the nature of the corona’s large-scale magnetic structure, including within the middle corona (e.g. Riley et al., 2019). Examples of forward modeling from these simulations are shown in Figure 12, which highlight how both broadband K-corona signatures as well as photo-excited coronal emission lines can be synthesized from MHD models. Such diagnostics can be used to extract information about the K- and F-corona from eclipse observations (Boe et al., 2021) as well as benchmark the temperature, densities, and charge-state distributions predicted by the MHD models through comparison to narrowband emission lines (Boe et al., 2022).

Other forward-modeling efforts specifically focused on structures within the middle corona include those of Goryaev et al. (2014), who developed a forward model to simulate the coronal emission of a streamer in EUV and visible-light, using assumed distributions of the electron density and temperature. The distribution parameters were determined by the solution that best fit EUV observations from SWAP and *Hinode*/EIS, and visible-light observations from the *Mauna Loa Mk4 Coronagraph*. The streamer-plasma temperature near the solar limb was found to be nearly isothermal from 1.2 – 2 $\mathrm{R}_{\odot }$, at $1.43\pm 0.08$ MK. They estimated the hydrostatic scale-height temperature from the determined density distribution and found it to be significantly higher, at $1.72\pm 0.08$ MK. They suggested that an outward plasma flow along the streamer could be the cause of the discrepancy. They estimated that more than 90% of the observed EUV emission from the streamer was due to collisional excitation, whereas in the background corona above ≈ 2 $\mathrm{R}_{\odot }$ resonant scattering may become comparable to collisional excitation in its contribution.

Del Zanna et al. (2018) developed a cylindrical-symmetry model that reproduced SOHO/UVCS observations of the H i Ly$\alpha $ and coronal lines between 1.4 – 3 $\mathrm{R}_{\odot }$ in quiescent streamers. The radial profile of the electron density was close to what was obtained from pB-measurements, and the ionization temperature was constant at 1.4 MK. The extrapolated densities at lower heights and the same temperature were successful in predicting the signal of the inner corona in near-infrared lines as measured during two solar eclipses in 2017 and 2019 by AIR-Spec, an airborne infrared spectrometer; see Madsen et al. (2019) and Samra et al. (2021).

### Modeling the Energetic Events

The basic picture of slow energy build-up through magnetic-field contortions and rapid energy release through magnetic reconnection is well established. However, the details of *how* that energy is released remain an area of active research. For CME energy storage and release alone, there are at least 26 review articles and ≥ 75 model articles spanning 18 physical mechanisms over the past two decades (Green et al., 2018 and references therein). A significant portion of that energy release is most clear in the middle corona, where CMEs experience the bulk of their acceleration (e.g. Bein et al., 2011; D’Huys et al., 2014).

Each of the numerous CME-mechanism models can produce predicted kinematic profiles for the resultant CME (height–time, speed–time, acceleration–time), which have characteristic shapes that can be altered by varying the dependencies in the model. For example, the torus instability model (Kliem and Török, 2006) can be modified with an upward-velocity perturbation whose duration can be modified – an approximation for continued energy release powering the acceleration – and not only does the acceleration–time profile peak at earlier times with a longer velocity perturbation, it can change from a single acceleration peak to having two acceleration peaks (Schrijver et al., 2008; Majumdar, Patel, and Pant, 2022).

As another example, the helical-kink instability tends to produce acceleration profiles with very strong jerks resulting from the magnetic-flux-rope twist exceeding a critical threshold of $448^{\circ}$ (e.g. Fan, 2016). In these two cases, and many others, the acceleration profiles differentiate themselves in the middle corona. A comprehensive summary of all of the physical mechanisms is beyond the scope of this article, but it has already been well covered by, e.g., Chen (2011) and Green et al. (2018).

Most of these models require magnetic reconnection to liberate the stored energy needed to accelerate CMEs and power their companion solar flares. Many reconnection models and observations also predict the formation of a large-scale plasma sheet associated with reconnection, extending from the low to middle corona. Although some models predict that reconnection itself occurs primarily in the inner corona (Forbes, Seaton, and Reeves, 2018), both of these models and observations of real events predict that upward-directed reconnection jets will dominate the dynamics of the middle corona in the wake of a CME (Yu et al., 2020). Sophisticated numerical models now appear to capture the dynamics of eruptive CME reconnection itself and the supra-arcade downflows (SADs) that often accompany this process (Shen et al., 2022). Since SADs often appear to originate from the middle corona (Savage and McKenzie, 2011), these new models help to explain one of the most important manifestations of energy release in this region in the wake of large eruptive events.

Importantly, both the limited observations we already have (Section 3) and the numerous models suggest that the middle corona is a key region for developing comprehensive understanding of CME energy release and acceleration. However, only with the next generation of high-sensitivity middle-corona observatories are we likely to obtain sufficient observations to develop comprehensive, data-constrained models of eruptions, that include the global-scale processes that govern the early evolution of these events.

### Global Coronal Models

Many studies have used PFSS extrapolations (Schatten, Wilcox, and Ness, 1969; Altschuler and Newkirk, 1969) to estimate the topology of the global coronal magnetic field, and hence consider the magnetic field of structures within the middle corona. For example, Goryaev et al. (2014) used such an extrapolation to estimate the magnetic structure of a coronal streamer, and Seaton et al. (2013a) that of a coronal fan.

One parameter in a PFSS is the source-surface height [$R_{\mathrm{ss}}$], which is the height at which magnetic-field lines become radial and are considered open. Many take the “default” value of the source surface to be $R_{\mathrm{ss}} = 2~\mathrm{R}_{\odot }$, although some studies have shown that a lower source-surface height may give a better fit to observations (e.g. Asvestari et al., 2019). Sarkar et al. (2019) combined observations from the large FOV of SWAP and the LASCO-C2/C3 to cover the whole middle-corona region, and by tracking the evolution of a cavity (EUV) into the three-part structure of the associated CME (visible-light) observed on 13 June 2010, they captured the kinematics of the eruption. By applying successive geometrical fits, they found that the cavity exhibited non-self-similar expansion in the inner and middle corona, below $2.2\pm 0.2~\mathrm{R}_{\odot }$, indicating a spatial scale for the radius of the source surface.

Recent work by Badman et al. (2022) used different parameters to constrain global models. The aim was to have different global models fit coronal holes on the disk as well as the neutral-line topology at the model’s outer boundary. Different observational datasets were used to then determine the accuracy of the fits. These included visible-light Carrington maps, EUV imaging, and *Parker Solar Probe* magnetic structures. The parameters could not be optimized simultaneously, meaning there is a trade off between measuring the coronal holes or the streamer-belt topology extending into the middle corona.

A non-potential magnetic-field model is a step up in complexity from a PFSS, allowing for free magnetic energy and electric currents within the volume. Meyer et al. (2020) investigated large-scale structures in the middle corona by comparing a data-driven, non-potential, global coronal magnetic-field model with EUV observations from SWAP. The lower boundary condition for the model was a global photospheric magnetic-flux transport simulation, which incorporated observed active-region magnetic-field data derived from the SDO/HMI-driven Advective Flux Transport (AFT) model (Upton and Hathaway, 2014), from September 2014 to March 2015. The initial condition for the model was a PFSS extrapolation for 1 September. The global coronal magnetic field was then evolved in time using a magneto-frictional relaxation method (van Ballegooijen, Priest, and Mackay, 2000; Mackay and van Ballegooijen, 2006), which produced a continuous series of non-potential equilibria in response to lower-boundary motions from the flux-transport simulation.

Meyer et al. (2020) considered the simulated coronal magnetic field from October 2014 onward, to allow sufficient time for the field to evolve away from its initially potential state. The model was found to reproduce the general structure of the global corona with a good degree of accuracy. Discrepancies between the observed and modeled corona typically occurred off the east solar limb, caused by active regions having emerged on the farside of the Sun, which cannot be incorporated into the model until they are observed on the near-side. The simulated corona was found to self-correct within a few days, but only after “late” active-region emergences were incorporated into the model. Meyer et al. followed the evolution of a particular coronal fan that was observed by SWAP extending into the middle corona over four Carrington rotations, from October 2014 to January 2015. The model was able to reproduce the observed structure of the fan, particularly when observed off the west limb. The model indicated that the magnetic structure underlying the fan changed from a streamer to a pseudostreamer configuration during its evolution.

Yeates et al. (2018) compared seven different global non-potential coronal magnetic-field models, which were all used to model the solar corona during the 20 March 2015 total eclipse. Included in the comparison were a magnetohydrostatic model (Bogdan and Low, 1986); non-linear force-free field models, including optimization (Wiegelmann, 2007), Grad–Rubin (Amari et al., 2013), force-free electrodynamics (Contopoulos, Kalapotharakos, and Georgoulis, 2011) and the time-evolving magneto-frictional method (Mackay and van Ballegooijen, 2006); and MHD models, including the AMR SIP–CESE solar-wind model (Feng et al., 2012) and MAS (Mikić et al., 1999) (where filament-channel locations based on this magneto-frictional simulation were used to energize the MAS model). All models produced static extrapolations extending toward or into the middle corona on the day of the eclipse, with the exception of the magneto-frictional model, which simulated a continuous time-evolution of the global corona from 1 September 2014 to 20 March 2015.

To evaluate their success, the plane-of-sky coronal structure of each model was compared with a stacked EUV image from SWAP and an Fe xiv 530.5 nm image of the corona during the eclipse, and sheared magnetic-field structures in the models were compared with filaments observed in an H$\alpha $ image from the Big Bear Solar Observatory. Yeates et al. (2018) found that the models showed general agreement in magnetic topology and the ratio of total to potential magnetic energy, but showed significant differences in electric current distributions. Static extrapolations were found to best reproduce active regions, while the time-evolving simulation could successfully recover filament-channel fields. The authors recommended overall that a hybrid approach may be most suitable, using static extrapolations that are energized by a simplified evolution model, such as the MAS/magneto-frictional hybrid example they presented.

Indeed, Mikić et al. (2018) produced a prediction of the global corona for the 21 August 2017 solar eclipse using the MAS model, energized by filament-channel information from a time-evolving magneto-frictional simulation in the months leading up to the eclipse. They compared the simulated corona with visible-light and EUV observations of the corona during the eclipse, finding that discrepancies between the model and simulations arose due to limitations in our current ability to observe the solar magnetic field, such as new active regions having emerged on the farside of the Sun.

Two observational constraints for global magnetic-field modeling are: Firstly, the open-field regions in the model should approximately correspond to coronal holes observed in emission, and secondly, that the magnitude of open flux from the model should match that determined from in-situ spacecraft. Linker et al. (2017) computed MHD and PFSS models from five different types of observatory magnetograms around July 2010. They found that for all combinations of maps and models, the models which had open-flux areas consistent with the observed coronal holes underestimated the interplanetary magnetic flux, and the models that matched the interplanetary magnetic flux had larger open-flux areas than the observed coronal holes, hence raising an open-flux problem.

Riley et al. (2019) investigated whether the “missing” open flux could be explained by adding flux to the polar regions, at latitudes too high to be resolved by ground-based observatories or Earth-based spacecraft. Through PFSS and MHD magnetic-field modeling, they showed that this additional polar flux could partially address the open-flux problem. These models were constructed to represent the 11 July 2010 total eclipse, so that plane-of-sky coronal structures could also be compared between the models and visible-light observations of the corona during the eclipse, where the global structure of the magnetic field becomes clear predominantly in the middle corona. Through this comparison, they concluded that the additional polar flux did not generate any new observational discrepancies, and indirectly demonstrated the value of middle-corona observations as an important constraint on global coronal models.

## Open Questions and a Strategy to Answer Them

The middle corona is a region of critical transitions straddling the inner and outer corona, to the point where it has occasionally been labeled *the transition corona* (e.g. Masson et al., 2014; Vourlidas et al., 2020; Golub et al., 2020). These transitions include the change from predominantly closed to open magnetic-field structures, and the change from low to high plasma-$\beta $ in specific regions. The middle corona is implicitly connected to both the inner and outer corona (and heliosphere by extension) through the continuation of the medium, and the bulk plasma and kinetic motions that pass from one to the other. Processes that occur within the middle corona can drive important effects in these regions and farther afield, including the Earth and other celestial bodies, especially as the result of its modulation of solar-wind outflow and CME kinematics.

The global-scale transitions that occur in the middle corona are neither ordered nor monotonic, and they depend strongly on the structures in which they occur. Plasma-$\beta $, for example, varies widely within the middle corona. In general, however, $\beta \ll 1$ in the inner corona, and magnetic field dominates plasma dynamics almost everywhere, while in the outer corona, $\beta $ can be variably above or below one, depending on local conditions. The location at which this transition occurs depends strongly on the type of structure observed and, in particular, these structures’ embedded magnetic field. Some observations (Seaton et al., 2021) suggest that large-scale dynamic processes in the middle corona can be driven by the gas dynamics of plasma flows, particularly in streamers.

Ultimately, the plasma kinetic energy dominates the magnetic-field structure in the super-Alfvénic flow regime beyond 10 – 20 $\mathrm{R}_{ \odot }$, as seen in *Parker Solar Probe* (Fox et al., 2016) in-situ data, even as plasma-$\beta $ varies across the unity threshold (Wexler et al., 2021). In *open*-field structures, such as plumes (e.g. DeForest, Plunkett, and Andrews, 2001) or streamers, it is believed that the release from low-$\beta $ dominance occurs mainly in the outer corona, where plasma can flow freely outwards. However, the quiet-Sun and active-region inner corona is dominated by closed-field structures with more complex topology, where under certain conditions the plasma pressure can overwhelm the magnetic pressure, such as along magnetic neutral lines in streamers (Vásquez, van Ballegooijen, and Raymond, 2003). The dominance of a particular force can have significant consequences for dynamic events. The dominance of magnetic pressure in the inner corona allows for the build up of magnetic energy, and field-aligned currents, which under certain conditions can be released as eruptions and flares. The relative transition from very low $\beta $ to a higher and variable role of gas pressure occurs largely in the middle corona. Understanding where the transitions occur will help us better understand the plasma dynamics and how flows and eruptions are influenced.

In spite of these important transitions, however, remote-sensing observations, both in radio and shorter wavelengths, have been insufficient to definitively characterize its global properties (see Section 3). Occasional instrumental off-points, eclipses (see, Section 3.1.2), and radio imaging have helped bridge the gap, but only intermittently.

Importantly, the primary methods used to observe the inner and outer corona already create an artificial boundary between these regions (Byrne et al., 2014). From an imaging perspective, the differing X-ray, EUV, and narrowband visible observations primarily sample line-of-sight *emission measure*, the emitting material at the temperature the passband samples, while broad-band visible coronagraph observations sample electron density (at all temperatures) along the line-of-sight.

Reconciling large-scale, multi-thermal, three-dimensional bright structures, such as solar eruptions, across multiple passbands is difficult (see, e.g., O’Hara et al., 2019). The absence of continuous and self-consistent observations, and an incomplete understanding of the underlying plasma properties, (see Section 5), has exacerbated the challenge of developing a deep understanding of the middle corona and its properties and behavior. We are left with several important questions that must be addressed to close this knowledge gap. In the following section we discuss a few of these key questions before presenting a broad strategy that could help to address them in Section 6.2.

### Open Questions Relating to the Middle Corona

#### Questions Concerning Transitions

##### What is the nature of middle-corona plasma, and how does its nature change from its inner to outer boundary?

The many transitions that occur within the middle corona include the change from predominantly *closed* to *open* magnetic-field structures and the change from low to high plasma-$\beta $ in quiet-Sun regions. These changes will vary throughout the region depending on the underlying coronal structures and plasma properties. The lack of comprehensive, systematic, and self-consistent observations through the region, in particular those that can provide density, temperature, and magnetic-field estimates, has impeded progress in determining where and how these transitions occur. Developing this understanding is critical to determining where and how processes such as solar-wind acceleration, ionization-state freezing-in, supersonic flows, and eruption and flow kinematic shaping occur.

##### Where does freeze-in occur in the middle corona? What can it tell us about the origins of solar-wind accelerated within middle corona structures?

The “freeze-in” altitude is the height at which charge states become fixed due to the plasma becoming too tenuous to sustain collisional ionization and recombination processes any further (Section 4.2). After this transition, ions become uncoupled from thermodynamic changes in the plasma and remain fixed. As a consequence, charge states are directly related to the heating and cooling experienced prior to freeze-in, making them an indirect diagnostic of coronal conditions. The height of freezing-in is still debated and can occur throughout the middle corona. New observations are required to constrain modeled plasma properties, which are used to derive freeze-in heights (Rivera et al., 2022a).

##### How does the magnetic topology of the corona transition from mostly closed to almost entirely open in the middle corona? What is the role of topology in determining dynamics within the region?

Outside of coronal holes, the inner corona is composed primarily of closed magnetic structures, while the outer corona is almost entirely radial, open magnetic field. The transition between these two regimes occurs entirely within the middle corona, but neither existing observations nor models have been sufficient to fully characterize how this transition occurs or the important role it plays in determining the dynamics that occur here. Increasingly, simulations (Higginson, 2016) and observations (Chitta et al., 2023) have revealed the ways in which the complex topology of this region and the interactions that occur in the S-web dictate structure embedded throughout the heliosphere, but much more work is needed.

#### Questions Concerning Outflow and Inflows

##### How does the evolving structure of the middle corona drive the structures that shape outflow into the solar wind?

Central to the understanding of solar-wind formation is the knowledge of the connection between the solar corona and the heliosphere (Viall and Borovsky, 2020). The heliospheric magnetic field is composed of an open field anchored in the photosphere, while lower in the corona the field is dominated by closed structures. The boundary between the open and closed fields, situated in the middle corona around 2 – 3 $\mathrm{R}_{ \odot }$, fluctuates and is distorted by physical processes on a broad range of scales (e.g. magnetic reconnection, eruptions, and continual flux emergence, Abbo et al., 2016). The feedback between these processes and the open/closed transition boundary is poorly understood, largely due to a lack of sensitivity and coverage in the middle-corona region, although the existence of such feedback may be inferred from the longevity of coronal holes, compared to the small-scale magnetic diffusion timescale at the photosphere. Understanding this feedback is critical for heliospheric studies since it determines how hot magnetized plasma enters interplanetary space. Furthermore, the open magnetic field and associated plasma are diverted from a purely radial direction by currents that produce a complex magnetic topology determined by photospheric evolution, prior dynamic events, and the field’s global structure (Newkirk, Altschuler, and Harvey, 1968; Wang, 1996; McComas et al., 2007; Yeates, Mackay, and van Ballegooijen, 2008). These deviations from the radial field have implications for the large-scale energy storage in the corona. These implications have yet to be fully explored because of the lack of observations in the middle-corona region.

##### What is the role of fine-scale plasma inhomogeneity perpendicular to the magnetic field?

The sonic point is considered an important benchmark for energy deposition within the corona, and it is believed to lie within the middle corona, potentially around 2 R_⊙_ (e.g. Cranmer, van Ballegooijen, and Edgar, 2007; Telloni, Giordano, and Antonucci, 2019). Energy deposition below or above this region is known to influence the properties of the outflowing solar wind, i.e. density, flow speed, temperature (e.g. Leer and Holzer, 1980). This has been confirmed in wave-driven solar-wind models, where amplifying the influence of different dissipation mechanisms, which predominantly act at different heights in the corona, leads to winds with different characteristics (Shoda, Yokoyama, and Suzuki, 2018). Although, knowledge of Alfvénic-wave propagation from the photosphere out into the heliosphere has long suffered from a lack of wave observations in the inner and middle corona that are able to provide meaningful constraints.

To this end, it has often sufficed to assume that the plasma throughout the corona has no plasma inhomogeneity perpendicular to the magnetic field, leading to simulations of coronal heating and wind acceleration focusing on the evolution of pure Alfvén waves. However, recent observations have demonstrated that the inner corona is highly structured, with over-dense magnetized plasma structures present in the quiet Sun and coronal holes (e.g. Thurgood, Morton, and McLaughlin, 2014; Morton, Weberg, and McLaughlin, 2019; Uritsky et al., 2021). This perpendicular inhomogeneity has been found to remain present out until at least to 14 R_⊙_ (DeForest et al., 2018), implying it must also be present in the middle corona. The presence of the inhomogeneities plays a critical role in wave propagation, preventing pure Alfvén modes. In their place, are surface Alfvén waves (Goossens et al., 2012), which are subject to resonances and enhanced phase mixing that pure Alfvén modes would not (Terradas, Goossens, and Verth, 2010; Pascoe, Wright, and De Moortel, 2010; Soler et al., 2019). Such phenomena concentrate wave energy to scales associated with the density structuring (Magyar and Van Doorsselaere, 2022). Previously, such mechanisms of wave dissipation were dismissed as unimportant for wave heating and acceleration. Hence, there are reawakened questions as to whether the structure of the inner and middle corona enables wave dissipation through resonances and phase mixing, and whether such physics is efficient enough to dissipate a meaningful fraction of energy before the sonic point.

##### What is the nature of the interface between the middle and outer corona? How do changes within the middle and outer corona propagate back to the Sun?

Inflows have been shown to interact with structures in the inner corona, including large-scale flows seen in EUV observations (Seaton et al., 2021), SADs seen in the wake of solar eruptions (Savage, McKenzie, and Reeves, 2012), and weaker inflows on many scales (Sheeley and Wang, 2014). Smaller or fainter downflows may also be ubiquitous in the less dynamic atmosphere, but could trigger eruptions through mechanisms such as magnetic breakout (e.g. Antiochos, DeVore, and Klimchuk, 1999). The exact nature of this interaction and the frequency of downflows are not fully known due to the weak signal in the far-field of EUV observations, and consequently a lack of observations. The lack of understanding of this feedback also leaves gaps in unified coronal-heliospheric models.

#### Questions Concerning Impulsive Events

##### What role does the middle corona play in CME acceleration? How does the middle corona influence the overall evolution of CMEs?

Impulsive CME acceleration is known to occur in the middle corona (Bein et al., 2011). Likewise, interactions within the middle corona can sometimes alter the trajectories of mature CMEs (D’Huys et al., 2017; Reva et al., 2017), potentially under the influence of the structure of magnetic field in the vicinity of the eruption (O’Hara et al., 2019) even to the point of preventing the CME from escaping at all (Thalmann et al., 2015; Alvarado-Gómez et al., 2018). However, the lack of consistent observations of the region presents a barrier to comprehensive understanding of how the forces and structures that emerge here manifest to shape the evolution of solar eruptions. By fully characterizing CME kinematics from the inner corona through the middle corona, we can ascertain how the background solar atmosphere interacts with the eruption, which forces are dominating, and perhaps understand the background solar conditions.

##### How does magnetic reconnection in the middle corona release stored magnetic energy to accelerate CMEs and heat the surrounding environment? What determines where this occurs?

Theoretical predictions suggest that the magnetic reconnection that powers eruptive solar flares should occur relatively low in the corona (Forbes, Seaton, and Reeves, 2018), but only a few observations, such as those by Yu et al. (2020) and Patel et al. (2020), have successfully isolated this location. Other manifestations of reconnection, such as SADs, can originate much higher – well into the middle corona – posing a mystery: what is the relationship between SADs and reconnection, and what do they have to teach us about one another? Likewise, other types of reconnection, such as magnetic breakout, may occur high above pre-eruptive structures (Lynch and Edmondson, 2013), potentially within the middle corona, but such processes have only rarely been observed. Recent observations of low coronal pseudostreamers have revealed the onset of CMEs via breakout reconnection at the 3D null point (Kumar et al., 2021). A similar mechanism is expected for the larger pseudostreamers in the middle corona, which requires further investigations. Still other types of CME, including so-called “stealth CMEs”, originate from unknown processes even higher in the middle corona (D’Huys et al., 2014), but may be driven by reconnection in streamers. Better observations of the middle corona are needed to provide insight into the role of magnetic reconnection in all of these disparate situations.

##### How do CME-driven waves and shocks influence the middle corona, particularly to accelerate particles? What can these tell us about CMEs themselves?

The interaction between CMEs and their associated shocks with the ambient middle corona is often studied from the viewpoint of how the CME kinematics are modulated by the ambient plasma conditions. However, the CME can also have important effects on the local surroundings. This can be manifest in many ways, and includes: through the aforementioned downflows generated in the wake of eruptions (SADs); the movement of surrounding structures, which can in turn force remote restructuring of the coronal magnetic structure and potentially generate sympathetic eruptions (Török et al., 2011); and the generation of SEPs from CME-driven shocks interacting with surrounding structures, such as streamers (Kong et al., 2017; Frassati, Mancuso, and Bemporad, 2020). Understanding these interactions is particularly important for the space-weather community.

### A Strategy to Maximize Our Understanding of the Middle Corona

Figure 2 presents a summary overview of the past, present, and near-term future of middle-corona observations, highlighting the patchwork nature of our coverage of this important region. The systematic observations of the solar disk and inner corona over the past few decades have been extraordinarily successful in addressing the longstanding questions that the instruments were optimized for. However, while middle-corona-optimized missions presently in development or proposed for the future are likely to lead to progress towards more systematic observations of the region, we remain a long way from the structured, well-coordinated observations needed to resolve the questions outlined in Section 6.1.

In particular, numerous studies, such as those of Byrne et al. (2014) and O’Hara et al. (2019), have demonstrated how difficult it is to associate the complex, 3D features of the middle corona that are observed in EUV with those observed in visible light. This is complicated by the huge disparity in coronal brightness across the region, necessitating complex image processing to coherently reveal structures and dynamic events that span the region. This observational gap must be closed.

To bridge this gap, UV and X-ray observations must be extended to greater heights, which can only be achieved through the development of high-sensitivity instrumentation, incorporating both low-noise detectors and strategies to obtain higher dynamic range observations. Missions such as SunCET (in development) and ECCCO (proposed) can serve as important pathfinders, so the technologies and strategies that follow them can lead to a generation of imagers that can fully connect the inner, middle, and outer coronae in a single FOV.

In contrast, the inner edge of the occulters required for visible-light and infrared coronagraphs must be reduced to lower heights. This can only be achieved with instrumentation incorporating improved stray-light rejection. The PROBA-3, *Aditya-L1/Visible Emission Line Coronagraph* (VELC: Prasad et al., 2017), UCoMP, and COSMO coronagraphs again serve as key pathfinders, but require complementary observations to extend the FOV to the outer edge of the middle corona. Future strategic planning is required to ensure the availability of co-temporal observations from all types of instruments discussed above. Additional targeted opportunities using low-cost platforms such as rockets, balloons, and eclipse observations can also fill important observational gaps.

Spectral observations are a crucial part of any middle-corona observation program and are required to help derive detailed understanding of the plasma properties of features captured in traditional images. Optical, UV, and EUV spectra provide unique diagnostics for densities, electron temperatures, ionization states, elemental compositions, kinetic temperatures and temperature anisotropies, Doppler-shift velocities along the line of sight, and velocities radially away from the Sun. Except for optical observations during eclipses, spectral observations of the middle corona have been largely limited to the UV spectra from UVCS, which operated from 1996 to 2013. These UVCS observations were severely hampered by SOHO’s low telemetry rate as well as a limited instantaneous spectral range and low sensitivity, all of which can easily be overcome by modern instruments and spacecraft. Closer integration of spectral and imaging observations, as designed for the *Large Optimized Coronagraphs for KeY Emission line Research* (LOCKYER) mission concept (Laming and Vourlidas, 2019), would greatly enhance the effectiveness of both.

While multiple radio facilities are available around the globe, there is no solar-dedicated radio instrument that provides true broadband, dynamic, imaging spectroscopy in the ≈ 0.4 – 1 GHz spectral range, which is critical to producing observations in support of key open questions about the middle corona, including CME initiation and acceleration, understanding the CME-accelerated electrons, and perhaps most importantly, to provide unique measurements of the evolving magnetic field of CMEs in the lower portion of the middle corona (≈ 1.2 – 2 $\mathrm{R}_{ \odot }$).

CMEs are faint and diffuse structures, which necessitate radio interferometers with a large number of antennas (several 10s to 100) to achieve sufficiently high-dynamic-range imaging (> 10^3^:1) and high surface-brightness sensitivity. In fact, these requirements for advancing radio studies of the middle-corona science toward the next stage already comprise one of the core objectives of the *Frequency Agile Solar Radiotelescope* (FASR) concept, which is envisioned to provide high-resolution, high-dynamic-range, and high-fidelity dynamic imaging spectroscopy over a wide frequency range from 0.2 – 20 GHz.

Structures throughout the corona are defined by the underlying magnetic fields; however, very few instruments can probe coronal magnetic fields at all, and only the *Upgraded Coronal Multi-channel Polarimeter* (UCoMP: Landi, Habbal, and Tomczyk, 2016) will be able to measure them anywhere close to the middle corona. Techniques to ascertain the coronal magnetic field are restricted to extrapolating magnetic fields from photospheric magnetograms and inferring them from density–temperature models. However, future instruments that leverage the Hanle effect, particularly in Lyman-$\alpha $ measurements (Raouafi et al., 2016a), will be able to much more directly ascertain the strength and orientation of the coronal magnetic field. Coupled with radio measurements, these observations can provide strong constraints on global models. Such new observations will significantly improve our ability to understand the topology, evolution, and global-scale effects of the middle corona’s complex magnetic field.

All of these individual measurements are important in their own right, but the middle corona in particular is a dynamic, three-dimensional environment that is very difficult to fully understood if only observed from a single, Earth-bound, perspective. Thus, developing true understanding requires $360^{\circ}/4\pi $ views of the Sun, including both the photospheric magnetic field and multiple lines of sight through coronal features and magnetic-field structures. Such observations, coupled to global magnetic-field models and advanced 3D reconstruction techniques (e.g. Plowman, 2021) would facilitate comprehensive understanding of the entire middle (and inner) corona. Such multi-perspective observations are required especially to characterize the highly structured and complex interfaces between middle-corona structures and the inner and outer coronae. Given the exotic solar orbits required to achieve these multi-perspective views, it is especially important to prioritize development of miniaturized instrumentation for multi-platform, deep-space constellations. The community must expedite the development such efforts via, e.g., expanded opportunities within NASA’s CubeSat and LCAS programs.

Trade studies are needed to prioritize limited resources in a coherent observing framework, balancing cost, risk, and criticality of observed physical parameters across the wide range of conditions in the middle corona. Some measurements can be made with distributed ground-based instrument networks, and some with miniaturized space-borne instruments, while others require significantly larger space-based investments. $360^{\circ}/4\pi $ observations – including out-of-ecliptic perspectives – should prioritize measurements that cannot be made from the Earth or Ecliptic perspective, or that facilitate significant research or space-weather forecasting progress using additional vantage points.

Coupling all of these new observations to global models is a major challenge, particularly determining how new magnetic-field and three-dimensional observations can be assimilated to provide model constraints. Advanced 3D reconstruction techniques and robust forward-modeling frameworks (Gibson et al., 2016) provide promising pathways to achieve better model/data integration. Further investments in models and model/data assimilation are required, and important lessons could be drawn from Earth and atmospheric science communities, which already have extensive data–model integration capabilities (Lahoz and Schneider, 2014).

## Conclusions

The middle corona, the region roughly spanning heliocentric heights from 1.5 to 6 $\mathrm{R}_{\odot }$, encompasses almost all of the influential physical transitions and processes that govern the behavior of coronal outflow. These transitions include the change from predominantly closed to open magnetic-field structures, and the change from low to high plasma-$\beta $ in specific regions. As a consequence of these transitions, the region is generally the location of primary solar-wind acceleration, ionization-state freezing-in, composition anomalies in long-lived structures, supersonic flow where the dynamical pressure exceeds the thermal pressure, and eruption and flow (and associated shock) kinematic shaping.

In spite of the important transitions that occur here, the middle corona remains poorly understood compared to both the inner and outer corona, primarily because it has been much more poorly observed than these regions. Remote-sensing observations, both in radio and shorter wavelengths, have been insufficient to definitively characterize its global properties. Occasional imaging opportunities, along with radio imaging and spectroscopic observations, have helped bridge the gap, but only intermittently and not consistently. Developing deep understanding of the large-scale multi-thermal structures from which the middle corona is predominantly composed has therefore proved difficult. We are left with numerous important questions that must be addressed to close this knowledge gap.

The object of this article was not to simply invent another naming convention, but rather to help define the new discovery space that is the middle corona. In particular, we aimed to highlight the deficiencies in our sporadic observations of the region and the numerous important open questions that follow from this. It is our hope the article will serve as a valuable summary and reference for what we know about important middle-coronal properties at the present time.

## Data Availability

Data used in the production of images in this manuscript are freely available from the instrument teams and data distribution nodes.
